# Pre‐Treatment Serum Prognostic Scores and Survival in Curatively Treated Laryngeal Cancer

**DOI:** 10.1002/lio2.70124

**Published:** 2025-03-22

**Authors:** Rhona Hurley, James Osbourne, Gareth J. Inman, David I. Conway, Claire Paterson, Catriona M. Douglas

**Affiliations:** ^1^ School of Cancer Sciences, Garscube Estate, University of Glasgow Glasgow UK; ^2^ Glasgow Head and Neck Cancer (GLAHNC) Research Group Glasgow UK; ^3^ Department of Otolaryngology/Head and Neck Surgery – Glasgow Royal Infirmary and Queen Elizabeth University Hospital Glasgow UK; ^4^ Cancer Research UK Scotland Institute Garscube Estate Glasgow UK; ^5^ School of Medicine, Dentistry and Nursing, University of Glasgow Glasgow UK; ^6^ Beatson West of Scotland Cancer Centre Glasgow UK

**Keywords:** cancer outcomes, head and neck cancer, laryngeal cancer

## Abstract

**Background:**

Laryngeal squamous cell cancer (LSCC) is a common head and neck cancer subtype, primarily linked to smoking and alcohol use. Despite declining incidence, survival outcomes have not improved. Prognostic scores, derived from blood‐based markers like neutrophil‐to‐lymphocyte ratio (NLR), platelet‐to‐lymphocyte ratio (PLR), lymphocyte‐to‐monocyte ratio (LMR), and systemic immune‐inflammation index (SIII), are increasingly recognized for their potential to predict survival outcomes and guide patient management.

**Methods:**

A retrospective analysis of 473 LSCC patients diagnosed in the West of Scotland (2014–2020) assessed the association of prognostic scores with overall survival (OS), cancer‐specific survival (CSS), and recurrence‐free survival (RFS). Patients were categorized into high‐ and low‐risk groups based on combined scores.

**Results:**

Low LMR and high‐risk scores were significantly associated with worse OS, CSS, and RFS. Nodal status and disease stage remained strong predictors. Combining clinicopathological variables with prognostic scores improved survival prediction.

**Conclusion:**

Prognostic scores are valuable tools for survival prediction in LSCC and, when combined with clinicopathological factors, may guide patient management. Further validation is warranted.

## Graphical abstract

This study evaluates the prognostic significance of systemic immune‐inflammatory markers in predicting survival outcomes for 473 patients with laryngeal squamous cell cancer (LSCC) treated curatively. Markers such as lymphocyte‐to‐monocyte ratio (LMR), neutrophil‐to‐lymphocyte ratio (NLR), and platelet‐to‐lymphocyte ratio (PLR) were significant predictors of overall survival, cancer‐specific survival, and recurrence‐free survival, with low LMR consistently associated with poor prognosis. Combining these markers with clinicopathological variables enhances predictive accuracy, providing a robust tool for personalized risk stratification in LSCC management.

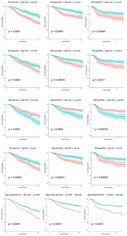

## 1 Introduction

Laryngeal squamous cell cancer (LSCC) is a common subtype of head and neck cancer. The main risk factors are smoking and alcohol use. In recent years, there has been a decrease in laryngeal cancer incidence [1, 2, 3, 4], which has been postulated to be related to a reduction in rates of cigarette smoking. Despite this, survival outcomes have not improved over the last several decades, with 5‐year survival in advanced disease remaining at around 60% [5, 6, 7]. In Scotland, 5‐year overall survival (OS) is 46% in data from 2014 to 2020 [8]. This compares unfavorably to Norway at 56.8% [9] and Sweden at 65% [10].

It is known that the interplay between immune and tumor cells is important within the tumor microenvironment (TME) [11], with infiltration of either pro‐tumorigenic or anti‐tumorigenic immune cells having an influence on survival and treatment outcomes [11, 12] However, the interaction of TME factors with patient factors, for example, age, gender and frailty is not well understood.

Previous research has examined the role of readily available, routine blood tests to calculate immune cell ratios—neutrophil‐to‐lymphocyte ratio (NLR), platelet‐to‐lymphocyte ratio (PLR), lymphocyte‐to‐monocyte ratio (LMR), and systemic immune‐inflammation index (SIII, platelets × neutrophils/lymphocytes). These can be calculated from a complete blood count (CBC). These have been used as prognostic predictors in various solid‐organ cancers, including head and neck, breast cancer and colorectal cancer to predict OS, disease‐free survival, and treatment response [13, 14, 15, 16]. Furthermore, they may have utility in predicting the risk of treatment complications, for example, high preoperative NLR has been associated with surgical complications in colorectal [17] and esophageal cancer [18], or adverse effects of oncological treatments, for example, radiation‐induced lung injury in lung cancer [19]. A combination of pre‐treatment NLR and pre‐treatment PLR has been found to be predictive of survival in non‐metastatic nasopharyngeal cancer, with better AUC (area under the curve) than these scores individually [20].

This study aims to explore the role of prognostic scores on OS, disease‐specific (CSS) and recurrence‐free survival (RFS) in laryngeal cancer. Secondly, this study aims to assess the value of combining serum prognostic scores in laryngeal cancer to predict survival outcomes.

## 2 Methods

### 2.1 Ethical Approval

UK Research Ethics Committee approval was obtained (IRAS project ID 333481).

### 2.2 Data Collection

This was a retrospective study of 473 patients diagnosed with LSCC in the West of Scotland from 2014 to 2020 treated curatively. Data were collated from the patients' electronic records. Follow‐up was calculated either to the date of death or the date of the last clinic review.

Routine blood test results, taken as work‐up prior to diagnostic biopsy and treatment, were recorded for those patients who had available results and were used to calculate serum prognostic scores. Results were within 1 month of the patients' date of diagnosis, prior to treatment commencement. Patients with documented active infection, for example, aspiration pneumonia, or other known conditions that may influence white cell count, for example, chronic lymphocytic leukemia, were excluded. The method of calculation of each serum prognostic score is shown in Table S1. Data on deprivation were collected, based on the area‐based Scottish Index of Multiple Deprivation [21] quintiles (1 = most deprived, 5 = least deprived). SIMD is an area‐based measure, calculated from domains such as income, crime, and employment [21]. Finally, patients were categorized based on modified frailty index‐5 (mFI‐5) [22] score into “Not frail,” “Moderately frail,” and “Severely frail” [23].

### 2.3 Statistical Analyses

Chi‐squared and Mann Whitney *U* tests were used to assess differences between groups (Table 2, Table S2). OS, CSS, and RFS were assessed from the date of the diagnostic cancer board meeting to the 5‐year follow‐up (where available, patients who did not have 5‐year follow‐up were censored). Cancer‐specific death was defined as death as a result of LSCC and its treatment. Recurrence was defined as the date of histological confirmation of recurrence or clinical confirmation if a biopsy was not performed. Cut‐off thresholds for “high” and “low” NLR, PLR, LMR, and SIII scores were calculated using receiver operating characteristic (ROC) curves, and optimal thresholds were subsequently determined using the Youden index ((maximum sensitivity + specificity − 1)) for each outcome.

Prognostic and clinicopathological markers that were significant in univariate survival analysis were included in a multivariate Cox proportional hazards model to determine if serum prognostic indicators are independent predictors of OS, CSS, and RFS. A *p* value of < 0.05 was considered significant, with the null hypothesis being that serum prognostic scores were not predictive of survival. Data analysis was performed using RStudio version 4.3.2 [24].

It was hypothesized that the performance of prognostic scores could be improved by creating a combination metric. Patients with high NLR, high PLR, and low LMR (as determined by the OS cutoffs described above) were grouped into a high‐risk category (HRG). Other patients were deemed low risk (LRG). These risk groups were then analyzed using univariate survival analysis, with statistical significance assessed using log‐rank testing. Median survival could not be calculated where 50% did not experience recurrence or disease‐related mortality.

Cox proportional hazards modeling was performed using risk groups. Models were assessed for OS, CSS, and RFS. Three types of models were evaluated: those with risk groups alone, those with clinicopathological variables alone, and those with both risk group and clinicopathological variables. Clinicopathological variables were included in the analysis for OS, CSS, and RFS if they were significant in univariate analysis. A linear predictor was derived from the Cox model and used to calculate time‐dependent ROC curves and area under the curve (AUC) values to assess the predictive value at 3 and 5 years of follow‐up.

## 3 Results

### 3.1 Patient Characteristics

A total of 473 patients were included. Relevant clinicopathological variables are presented in Table 1. Most patients were male (79.9%) and under 65 years of age (51.4%). Nearly half of the patients (53.4%) presented with advanced disease (AJCC stage 3 and 4), and 48.8% had supraglottic cancer. Most patients were treated with radiotherapy (43.3%) or single‐modality surgical treatment (37.2%). Recurrence was observed in 26.4% of the patients. The mean follow‐up time was 54.6 months. 78.6% of patients were at least “moderately frail”, and 71.5% lived in the two most deprived quintiles as categorized by the Scottish Index of Multiple Deprivation (SIMD).

**TABLE 1:** Demographic and clinical characteristics of all 473 patients.

Variables	*N* = 473 (%)#
Age	
< 65	243 (51.4%)
65–74	167 (35.3%)
> 75	63 (13.3%)
Sex	
Male	378 (79.9%)
Female	95 (20.1%)
ASA grade	
1	13 (2.7%)
2	112 (23.7%)
3	238 (50.3%)
4	17 (3.6%)
Missing data	92 (19.5%)
Tumor stage	
T1	125 (26.4%)
T2	109 (23%)
T3	143 (30.2%)
T4	96 (20.3%)
Nodal stage	
N0	383 (81%)
N1	25 (5.3%)
N2	55 (11.6%)
N3	10 (2.1%)
Metastasis stage	
M0	472 (99.8%)
M1	1 (0.2%)
AJCC stage	
1	127 (26.8%)
2	93 (19.7%)
3	124 (26.2%)
4	129 (27.2%)
Subsite involvement	
Supraglottic	231 (48.8%)
Glottic	182 (38.5%)
Subglottic	3 (0.63%)
Transglottic	54 (11.4%)
Indeterminate	3 (0.63%)
Treatment modality	
Surgery only	176 (37.2%)
Radiotherapy only	205 (43.3%)
Surgery and radiotherapy	57 (12.1%)
Chemoradiotherapy	24 (5.1%)
Trimodality	11 (2.3%)
Recurrence	
Yes	125 (26.4%)
No	348 (73.6%)
WHO performance status PS	
0	227 (48.0%)
1	185 (39.1%)
2	57 (12.1%)
3	4 (0.8%)
Frailty	
Not frail	101 (21.4%)
Moderately frail	241 (51%)
Severely frail	131 (27.6%)
Scottish Index of Multiple Deprivation (SIMD) quintile	
1 (most deprived)	226 (47.8%)
2	112 (23.7%)
3	49 (10.4%)
4	41 (8.7%)
5 (least deprived)	38 (8.0%)
Not found	7 (1.5%)

Abbreviations: AJCC = American Joint Committee on Cancer, ASA = American Society of Anesthesiology, WHO=World Health Organization.

### 3.2 Calculation of Optimal Thresholds and Univariate Survival Analysis

**FIGURE 1:**
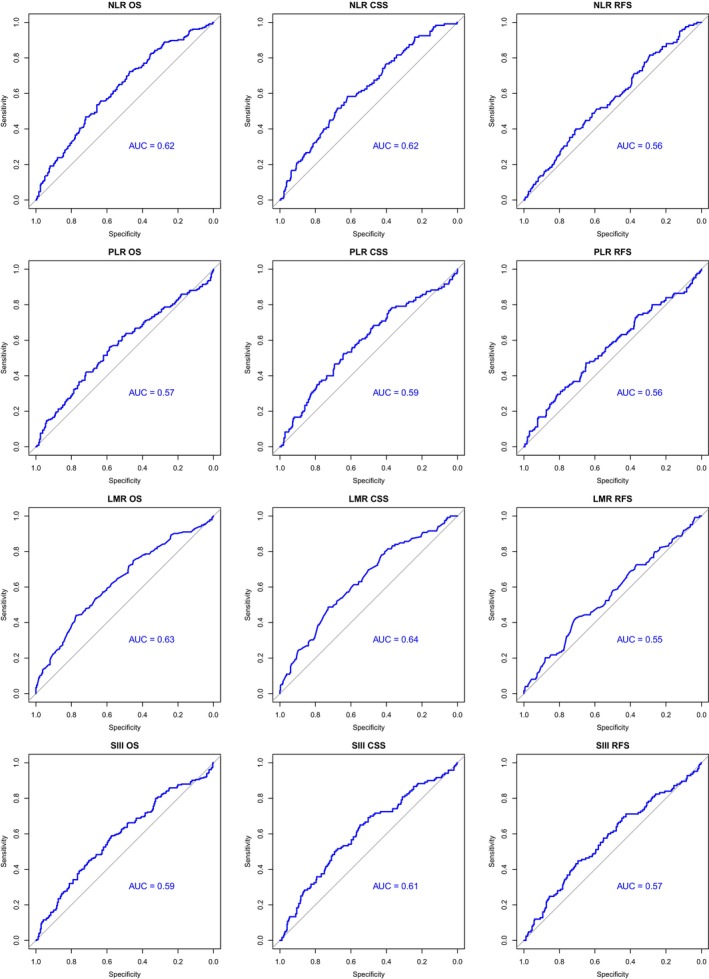
Receiver operating curves (ROC) for NLR, PLR, LMR, and SIII for OS, CSS, and RFS with area under the curve (AUC) annotated.

A summary of the results for each prognostic marker is shown in Table S2 but is described below. ROC curves for OSS/CSS/RFS for NLR, PLR, LMR, and SIII are shown in Figure 1. Kaplan–Meier curves (OS/CSS/RFS) are shown for prognostic markers in Figure S1.

- NLR

For OS, the NLR threshold was 2.34, to give a sensitivity of 0.72 and specificity of 0.47, and an AUC of 0.62. Two hundred and ninety‐six patients had high NLR, and 179 had low NLR by this threshold. For CSS, the NLR threshold was 2.98, with sensitivity of 0.58 and specificity of 0.62, with an AUC of 0.62. Two hundred and seven patients had high NLR, and 268 had low NLR by this threshold. For RFS, the optimal threshold was 3.63, with sensitivity of 0.4, specificity of 0.71, and AUC of 0.56. By this threshold, 151 patients had high NLR, and 322 had low NLR.

High NLR was found to predict worse OS (*p* < 0.0001) with a median survival of 55 months, compared with 80 months in the low NLR group. Similarly, high NLR predicted poorer CSS *p* (< 0.0001) with a median survival of 94 months in the high NLR group. Median survival could not be calculated for the low NLR group as 50% had not experienced cause‐specific mortality. High NLR predicted worse RFS on univariate analysis (*p* = 0.00081).

b PLR

For OS, the PLR threshold was 142.5, with a sensitivity of 0.58 and specificity of 0.59, with an AUC of 0.57. By this threshold, 231 patients had high PLR and 243 had low PLR. For CSS, the PLR threshold was 152.1, with a sensitivity of 0.52 and a specificity of 0.62, with an AUC of 0.59; 191 patients had high PLR and 283 had low PLR by this threshold. For RFS, the threshold was 156.2, with a sensitivity of 0.47, a specificity of 0.65, and an AUC of 0.56. By this threshold, 181 patients had high PLR, and 292 had low PLR.

High PLR was found to predict worse OS (*p* = 0.0052), with a median survival of 56 months, compared with 75 months in the low PLR group. High PLR predicted poorer CSS (*p* = 0.00078), with a median survival of 94 months in the high PLR group. Median survival could not be calculated for the low PLR group as 50% had not experienced cause‐specific mortality. PLR predicted worse RFS (*p* = 0.0017).

c LMR

For OS, the LMR threshold was 2.33, with a sensitivity of 0.44 and specificity of 0.78, with an AUC of 0.63. For CSS, the LMR threshold was also 2.33, with a sensitivity of 0.49 and specificity of 0.73, with an AUC of 0.64. Three hundred and eighteen patients had high LMR and 157 had low LMR by this threshold. For RFS, the LMR threshold was 2.21, with a sensitivity of 0.42 and specificity of 0.72, with an AUC 0.55. By this threshold, 310 patients had high LMR and 162 had low LMR.

Low LMR was found to predict worse OS (*p* < 0.0001), with median survival of 49 months, compared with 78 months in the high LMR group. Low LMR predicted poorer CSS (*p* < 0.0001), with median survival of 77 months in the low LMR group compared with 117 in the high LMR group. Low LMR predicted worse RFS on univariate analysis (*p* = 0.00016).

d SIII

For OS, the SIII threshold was 675.12, with a sensitivity of 0.59 and a specificity of 0.57, with an AUC of 0.59. For CSS, the SIII threshold was also 675.12, with a sensitivity of 0.65 and a specificity of 0.55, with an AUC of 0.61. Two hundred and forty patients had high SIII and 233 had low SIII. For RFS, the SIII threshold was 969.7, with a sensitivity of 0.44 and a specificity of 0.7, with an AUC of 0.57. By this threshold, 311 patients had high SIII, and 161 had low SIII.

High SIII was found to predict worse OS (*p* = 0.00097), with a median survival of 55 months compared with 78 months in the low SIII group. High SIII predicted worse CSS (*p* = 0.00021), with a median survival of 101 months in the high SIII group. Median survival could not be calculated for the low SIII group as 50% had not experienced cause‐specific mortality. High SIII predicted poorer RFS in univariate analysis (*p* = 0.00059).

Differences between clinical characteristics of patients with high and low OS cut‐off scores are explored in Table S3. There was a statistically significant difference in age (*p* = 0.02) and gender (*p* = 0.002) for LMR. There was a difference in stage at presentation between high and low survival cut‐off for SIII (*p* = 0.0004), LMR (0.03) and NLR (*p* = 0.003), WHO performance status for LMR (*p* = 0.02) and SIII (*p* = 0.02), frailty for LMR (*p* = 0.0001) and SIII (*p* = 0.0157), nodal status for NLR (*p* = 0.03) and SIII scores (*p* = 0.0002), and subsite (*p* = 0.01) for SIII scores.

e Survival analysis of clinicopathological factors to determine inclusion in multivariate analysis

The effect of age on survival was analyzed using a Cox proportional hazards model. This was statistically significant for OS (*p* = 0.0007), but not for CSS (*p* = 0.15) or RFS (*p* = 0.7).

Kaplan–Meier curves for categorical variables are shown in Figure S2. For OS, subsite involvement, stage, nodal status, smoking status, performance status, frailty, and stage were all statistically significant predictors of survival. For CSS, site, stage, nodal status, and performance status were statistically significant predictors of survival. For RFS, stage, nodal status, and performance status were statistically significant.

### 3.3 Multivariate Survival Analysis

This analysis included variables that were significant in univariate analysis (see above). The Cox proportional hazards model output for OS, CSS, and RFS is shown in Table S4. Low LMR and high NLR indicated worse OS in multivariate analysis. Other significant predictors included age, nodal status, and WHO performance status (PS) 1 or 2, showing improved survival outcomes.

For CSS, low LMR remained the only significant prognostic marker, indicating worse survival. Similar to OS, nodal status and WHO performance status (PS) 1 or 2 were associated with significantly worse CSS, as well as subglottic and transglottic disease.

For RFS, Low LMR was the only statistically significant prognostic marker. PS 1 and Nodal stage N2 and N3 remained significant in multivariate analysis.

### 3.4 Assessment of Combination Score to Form Risk Groups

One hundred and fifteen (24.3%) were classified as high risk (HRG: high NLR, high PLR, low LMR), and 358 (75.6%) as low risk (LRG). Detailed clinical characteristics of these groups are shown in Table 2, with significant differences in sex (*p* = 0.02), PS (*p* = 0.02), and frailty status (*p* = 0.001).

**TABLE 2:** Examining the different clinical characteristics of risk groups as characterized by NLR, PLR, and LMR.

Characteristic	High‐risk group	Low‐risk group	*p*
	*N* = 115 (24.3%)	*N* = 358 (75.6%)	
Mean age	65.1	63.6	0.139
Gender			
Male	101 (87.8%)	277 (77.4%)	
Female	14 (12.2%)	81 (22.6%)	0.02*
Smoking			
Never	11 (9.6%)	30 (8.4%)	
Ex‐smoker	46 (40%)	113 (31.6%)	
Current	58 (50.4%)	215 (60%)	0.18
Stage			
Early	44 (38.3%)	176 (49.2%)	
Advanced	71 (61.7%)	182 (50.8%)	0.053
Subsite			
Glottic	40 (34.8%)	142 (39.7%)	
Supraglottic	55 (47.8%)	176 (49.2%)	
Subglottic	1 (0.9%)	2 (0.6%)	
Transglottic	18 (15.7%)	36 (10.1%)	
Indeterminate	1 (0.9%)	2 (0.6%)	0.52
Nodal status			
N0	87 (75.7%)	296 (82.7%)	
N1	7 (6.1%)	18 (5%)	
N2	18 (15.7%)	37 (10.3%)	
N3	3 (2.6%)	7 (2%)	0.39
Performance status			
0	46 (40%)	181 (50.6%)	
1	47 (40.9%)	138 (38.5%)	
2	19 (16.5%)	38 (10.6%)	
3	3 (2.6%)	1 (0.3%)	0.02*
Treatment			
RT only	48 (41.7%)	157 (43.9%)	
Surgery only	41 (36.7%)	135 (37.7%)	
Surgery + RT	15 (13%)	42 (11.7%)	
CRT	6 (5.2%)	18 (5%)	
Trimodality	5 (4.4%)	6 (1.7%)	0.55
Deprivation^			0.69
1	55 (47.8%)	171 (47.8%)	
2	24 (20.9%)	88 (24.6%)	
3	16 (13.9%)	33 (9.2%)	
4	8 (7%)	33 (9.2%)	
5	10 (8.7%)	28 (7.8%)	
Not found	2 (1.7%)	5 (1.4%)	
Frailty			0.001**
Not frail	18 (15.6%)	83 (23.2%)	
Moderately frail	50 (43.5%)	191 (53.4%)	
Severely frail	47 (40.9%)	84 (23.4%)	
Median survival in months			
OS	48	70	
CSS	74	119	
RFS	61	112	

*Note:* High‐risk group was defined as high NLR AND high PLR AND low LMR. Cutoffs used to generate groups were the overall survival cutoffs. Chi‐square tests were used to assess differences between groups, except for mean age in which differences between groups were assessed using the Mann Whitney *U* test due to non‐parametric distribution. ^Deprivation was measured by SIMD quintiles. *p* values < 0.05 were defined as significant (*), with *p* values < 0.001 highly significant (**).

The risk stratification was statistically significant in the univariate survival analysis for OS, CSS, and RFS (Figure S1). Therefore, multivariate analysis was performed, incorporating this stratification with relevant clinical factors. Results are shown in Table 3 for OS, CSS, and RFS. In all analyses, the high‐risk group (HRG) remained an independent predictor of poor survival. The HRG confers a 1.62 hazard ratio for overall mortality (*p* < 0.001). Age, current smoking, advanced disease, nodal status, and PS remain significant predictors of overall mortality.

**TABLE 3:** Multivariate survival analysis by risk groups.

Variable	Hazard ratio (HR)	95% CI	*p*
Overall survival—Risk groups			
Age	1.03	1.02–1.05	< 0.001**
Performance status (ref = PS 0)			
1	1.62	1.19–2.20	0.002*
2	1.70	1.04–2.76	0.034*
3	1.39	0.41–4.76	0.6
Smoking (ref = never smoked)			
Ex	1.33	0.72–2.44	0.4
Current	1.87	1.02–3.41	0.042*
Stage (ref = early)			
Advanced	1.46	1.03–2.06	0.032*
Subsite (ref = glottic)			
Supraglottic	1.19	0.85–1.65	0.3
Subglottic	3.28	0.76–14.1	0.11
Transglottic	1.15	0.72–1.83	0.6
Indeterminate	2.99	0.89–9.99	0.076
Nodal status (ref = N0)			
N1	1.12	0.62–2.02	0.7
N2	2.36	1.60–3.47	< 0.001**
N3	4.30	1.99–9.28	< 0.001**
Frailty (ref = not frail)			
Moderately frail	1.09	0.73–1.63	0.7
Severely frail	1.04	0.65–1.69	0.9
Risk group (ref = low risk)			
High risk	1.62	1.21–2.18	< 0.001**
Cancer‐specific survival—Risk groups			
Performance status (ref = PS 0)			
1	2.17	1.42–3.30	< 0.001**
2	2.20	1.26–3.83	0.006*
3	0.63	0.08–4.78	0.7
Stage (ref = early)			
Advanced	1.48	0.89–2.44	0.13
Subsite (ref = glottic)			
Supraglottic	1.31	0.81–2.12	0.3
Subglottic	5.79	1.31–25.7	0.021*
Transglottic	1.88	1.01–3.50	0.045*
Indeterminate	3.56	0.79–16.0	0.10
Nodal status (ref = N0)			
N1	1.33	0.62–2.88	0.5
N2	2.14	1.30–3.52	0.003*
N3	4.46	1.85–10.8	< 0.001**
Risk group (ref = low risk)			
High risk	1.95	1.33–2.86	< 0.001**
Recurrence‐free survival—Risk groups			
Performance status (ref = PS 0)			
1	1.74	1.18–2.56	0.006*
2	1.30	0.73–2.32	0.44
3	1.82	0.43–7.71	0.4
Stage (ref = early)			
Advanced	1.06	0.70–1.60	0.8
Nodal status (ref = N0)			
N1	1.14	0.48–2.70	0.8
N2	2.18	1.29–3.67	0.004*
N3	3.05	1.08–8.65	0.036*
Risk group (ref = low risk)			
High risk	2.08	1.42–3.06	< 0.001**

*Note:* *p* values < 0.05 were defined as significant (*), with *p* values < 0.001 being highly significant (**).

Abbreviations: CI = confidence interval, HR = Hazard ratio.

The HRG confers a 1.95 hazard ratio for disease‐specific mortality (*p* < 0.001). Subglottic disease, transglottic disease, nodal status N2/N3, PS 1, and PS 2 are also statistically significant predictors of disease‐specific mortality.

The HRG confers a 2.08 hazard ratio for recurrence (*p* < 0.001). PS 1 and nodal status remain significant predictors of recurrence on multivariate analysis.

### 3.5 Predictive Modeling of Risk Groups and LMR

Summary results for AUC at 3 and 5 years are shown in Table 4. As LMR was predictive on multivariate analysis in OS, CSS, and RFS, this was assessed alongside the risk group categorization. The combination of clinicopathological factors and risk group/LMR together provided superior AUC for OS, CSS, and RFS. However, clinicopathological factors alone outperformed the risk group/LMR alone.

**TABLE 4:** Area under the curve analysis for survival models.

Model	Survival type	AUC
Risk group alone	OS	3 year 0.56
		5 year 0.58
LMR alone	OS	3 year 0.55
		5 year 0.60
Clinicopathological factors alone (age, smoking, site, stage, nodal status, PS, frailty)	OS	3 year 0.73
		5 year 0.78
Risk group + Clinicopathological factors	OS	3 year 0.74
		5 year 0.79
LMR + Clinicopathological factors	OS	3 year 0.73
		5 year 0.79
Risk group alone	CSS	3 year 0.58
		5 year 0.60
LMR alone	CSS	3 year 0.58
		5 year 0.62
Clinicopathological factors alone (site, stage, nodal status, PS)	CSS	3 year 0.77
		5 year 0.78
Risk group + Clinicopathological factors	CSS	3 year 0.78
		5 year 0.79
LMR + Clinicopathological factors	CSS	3 year 0.78
		5 year 0.8
Risk group alone	RFS	3 year 0.59
		5 year 0.64
LMR alone	RFS	3 year 0.59
		5 year 0.66
Clinicopathological factors alone (stage, nodal status, PS)	RFS	3 year 0.67
		5 year 0.70
Risk group + Clinicopathological factors	RFS	3 year 0.69
		5 year 0.75
LMR + Clinicopathological factors	RFS	3 year 0.69
		5 year 0.76

*Note:* This table examines the AUC for OS/CSS/RFS for risk group/LMR as predictor alone, clinicopathological factors alone, and combined. Combined, they provide the best AUC indicating predictive ability; however, clinicopathological factors alone are stronger predictors than risk group alone.

## 4 Discussion

This study explores the role of serum prognostic markers as predictors of survival in a cohort of patients with LSCC. While numerous studies have investigated the prognostic value of blood markers, findings have often been conflicting. This study aimed to use a combination score to risk stratify patients and to assess its predictive value for OS, CSS, and RFS in combination with clinicopathological factors. The HRG modestly improved the outcome prediction of OS, CSS, and RFS in conjunction with clinicopathological variables.

The TME has been strongly implicated in cancer pathogenesis, progression, and metastasis. Tumor infiltrating lymphocytes (TILs), known to be protective in laryngeal cancer and oropharyngeal cancer [25, 26], have spurred interest in serum prognostic scores derived from white cell counts as surrogate markers of the TME. In particular, LMR has been shown to correlate with levels of TILs [27, 28]. This study describes the association between these prognostic scores (NLR, PLR, LMR, and SIII) in predicting OS, CSS, and RFS. On univariate analysis, all were significantly associated with OS, CSS, and RFS. However, on multivariate analysis, only low LMR was significantly associated with poorer OS, CSS, and RFS, while high NLR was significantly associated with poorer OS.

This study builds on previous findings regarding serum prognostic scores in laryngeal cancer, which have shown conflicting results. Notably, two meta‐analyses identified NLR as an independent predictor of survival [29, 30]. However, there are some studies that do not demonstrate this relationship with mortality but with other outcomes such as recurrence [31]. In our cohort, high NLR is predictive of poorer OS on univariate and multivariate analysis but not predictive of poorer CSS/RFS.

The interaction of patient factors with prognostic scores may contribute to some of the variability across studies. In this cohort, frailty was prevalent, with over 75% of patients classified as moderately or severely frail when measured by the mFI‐5. Frailty has been examined in head and neck cancer as a whole, with frailty rates of 32%–36% [32, 33]. Patients with laryngeal cancer may be frailer than undifferentiated head and neck cancer patients due to association with older age and smoking. However, frailty in this cohort is much higher (> 75%) than in a previous study in which 7.2% of all HNSCC patients were frail [34], although some differences will be due to the difference in definitions of frailty. NLR is associated with frailty in the general population [35] and frailty has been shown to be associated with increased NLR in colorectal cancer patients [23]. Low LMR has been associated with increasing comorbidity and frailty [35]. This is mirrored in our results. Therefore, it is likely that patient factors such as frailty and comorbidity have an impact on serum prognostic scores; this may have an impact on generalizability to other HNSCC subsites.

Serum prognostic markers, including NLR, PLR, LMR, and SIII, have been shown to be associated with occult lymph node metastasis in HNSCC, with NLR being the strongest predictor in combination with factors such as tumor subsite and stage [36]. As occult nodal metastasis in LSCC occurs in around 20.5% [37], pretreatment prognostic scores may help guide decisions on elective neck treatment for node‐negative disease. Currently, in node‐negative disease, elective treatment to the neck is offered when the risk of occult metastasis (around 15%–20%) [37, 38, 39] outweighs the risk of increased morbidity from treatment. Prospective validation could establish the value of these markers in predicting occult metastasis risk.

Potential future directions for the use of serum prognostic scores could include prediction of complications after surgery, as has been demonstrated in colorectal surgery [17, 40, 41]. NLR has been shown to predict pharyngocutaneous fistula after laryngectomy [42, 43], potentially allowing early intervention, for example, salivary bypass tube insertion or use of pedicled flaps at the time of surgery. One study has noted that pre‐treatment high LMR and an increase in LMR during radiotherapy treatment are predictive of outcomes [44]. It may be possible to predict the likelihood of (chemo) radiotherapy toxicity, although one study demonstrated no association of toxicity with NLR in patients with HNSCC [45].

Combinations of prognostic scores have been used to improve their predictive accuracy. In non‐metastatic nasopharyngeal cancer, combined NLR/PLR was demonstrated to have increased accuracy in predicting survival compared to individual measurements of these scores [20]. As NLR, PLR, and LMR can be derived from a full blood count, a single low‐cost test, this study proposes categorizing patients based on all three markers into risk groups. An AUC of > 0.7 indicates good predictive ability and > 0.8 indicates excellent predictive ability [46]. Neither LMR nor risk groups reach this threshold alone, but combining this score with clinicopathological factors increases predictive ability to 75%–80% in the case of 5‐year OS, CSS, and RFS (Table 4).

Due to the mixed results in the literature regarding the prognostic ability of NLR, PLR, and LMR [29, 47, 48, 49], as well as the relationship of prognostic scores with patient factors like frailty, it may be that combining these scores into a composite value will improve predictive ability and reduce inter‐study heterogeneity. Given the current evidence for the predictive value of NLR for fistula after laryngectomy [42, 43], retaining this information as a composite score could mean that risk group categorization could have multiple applications. Future work will focus on the validation of this risk group approach in other laryngeal cancer cohorts, in other subsites and for the prediction of treatment outcomes. It may be that using the CSS or RFS cut‐off instead of the OS cut‐off to delineate the risk groups may improve performance. If validated, these risk group scores could be used to inform discussions with patients with regard to treatment escalation, particularly in the case of clinically node‐negative T3/T4 disease, or for the prediction of complications after surgery.

The limitations of this study include the need for validation of the serum prognostic score cut‐offs and risk group stratification in other LSCC cohorts and in other HNSCC subsites to assess generalizability to broader populations. Information on corticosteroid use was not collected, which could potentially impact blood results. While it is unlikely that corticosteroid use was widespread (except in cases of airway obstruction), this factor cannot be accounted for in our analysis.

Our study spans a single cancer network, meaning lab results were generated by different laboratories. However, as ratios were used to calculate serum prognostic scores, we do not anticipate this to have a significant impact on the results. In our center, pre‐treatment CRP is not routinely measured, so data on mGPS were not included in this study.

Retrospective data collection relies on electronic patient records, which is likely to lead to underestimation of exclusion criteria (e.g., active infection) in this cohort. Finally, as prognostic scores were based on a single measurement, they represent a snapshot in the disease course and were not collected at a single standardized time point—most were gathered before biopsy. A prospective study would allow for collection at a defined, consistent time point and enable serial measurement throughout treatment. Future studies should aim to examine dynamic changes in these scores over the course of treatment through prospective data collection.

## 5 Conclusion

Prognostic scores derived from the readily available CBC are predictors of OS, CSS, and RFS in laryngeal cancer, in particular, the lymphocyte‐monocyte ratio. Their utility can be increased in combination with clinicopathological variables to improve predictive value. This approach could assist in decision‐making regarding patient management if this approach can be further validated. It is notable that performance status remains important in multivariate analysis, and this raises the question of the impact of patient factors on serum prognostic scores. Further research will focus on the relationship between prognostic scores and treatment outcomes, and contextualizing results with transcriptomic analysis of historic patient tissue samples from this cohort.

## Conflicts of Interest

The authors declare no conflicts of interest.

## Supporting information


**Data S1.** Supporting Information.

## Data Availability

Anonymized patient data and associated code can be made available upon reasonable request.

## References

[1] S. E. Mousavi , M. Ilaghi , A. Aslani , M. Najafi , Z. Yekta , and S. A. Nejadghaderi , “Laryngeal Cancer Incidence Trends in the United States Over 2000–2020: A Population‐Based Analysis,” Archives of Public Health = Archives Belges de Sante Publique 82, no. 1 (2024): 106, 10.1186/s13690-024-01333-1. 38987838 PMC11234729

[2] R. L. Siegel , K. D. Miller , H. E. Fuchs , and A. Jemal , “Cancer Statistics, 2022,” CA: A Cancer Journal for Clinicians 72 (2022): 7–33, 10.3322/caac.21708. 35020204

[3] R. L. Siegel , K. D. Miller , and A. Jemal , “Cancer Statistics, 2015,” CA: A Cancer Journal for Clinicians 65 (2015): 5–29, 10.3322/caac.21254. 25559415

[4] European Union , “ECIS—European Cancer Information System,” 2024.

[5] E. Francis , N. Matar , N. Khoueir , C. Nassif , C. Farah , and A. Haddad , “T4a Laryngeal Cancer Survival: Retrospective Institutional Analysis and Systematic Review,” Laryngoscope 124 (2014): 1618–1623, 10.1002/lary.24557. 24338374

[6] M. M. Li , S. Zhao , A. Eskander , et al., “Stage Migration and Survival Trends in Laryngeal Cancer,” Annals of Surgical Oncology 28, no. 12 (2021): 7300–7309, 10.1245/s10434-021-10318-1. 34263369 PMC9013009

[7] P. Divakar and L. Davies , “Trends in Incidence and Mortality of Larynx Cancer in the US,” JAMA Otolaryngology. Head & Neck Surgery 1 (2022): 34–41. 10.1001/jamaoto.2022.3636 PMC9673027 36394832

[8] C. McCann , R. Hurley , J. McGovern , K. Knight , N. J. W. Rattray , and C. M. Douglas , “Frailty and the Survival Outcomes of Patients With Laryngeal Squamous Cell Cancer,” Head & Neck (2024), 10.1002/hed.27951. 39578701

[9] J. Brandstorp‐Boesen , R. S. Falk , M. Boysen , and K. Brøndbo , “Impact of Stage, Management and Recurrence on Survival Rates in Laryngeal Cancer,” PLoS One 12, no. 7 (2017): e0179371, 10.1371/journal.pone.0179371. 28708883 PMC5510803

[10] R. Blomkvist , L. Marklund , L. Hammarstedt‐Nordenvall , E. Gottlieb‐Vedi , A. Mäkitie , and B. Palmgren , “Treatment and Outcome Among Patients With Laryngeal Squamous Cell Carcinoma in Stockholm—A Population‐Based Study,” Laryngoscope Investig Otolaryngol 8 (2023): 441–449, 10.1002/lio2.1034. PMC10116984 37090883

[11] S. M. Y. Chen , A. L. Krinsky , R. A. Woolaver , X. Wang , Z. Chen , and J. H. Wang , “Tumor Immune Microenvironment in Head and Neck Cancers,” Molecular Carcinogenesis 59 (2020): 766–774, 10.1002/mc.23162. 32017286 PMC7282929

[12] A. Elmusrati , J. Wang , and C. Y. Wang , “Tumor Microenvironment and Immune Evasion in Head and Neck Squamous Cell Carcinoma,” International Journal of Oral Science 13, no. 1 (2021): 24, 10.1038/s41368-021-00131-7. 34341329 PMC8329257

[13] C. W. Luan , L. T. Kuo , Y. T. Wang , et al., “Utility of Modified Glasgow Prognostic Score for Head and Neck Squamous Cell Carcinoma: Systematic Review and Meta‐Analysis,” Head & Neck 45 (2023): 1856–1867, 10.1002/hed.27397. 37161915

[14] M. S. Johnstone , S. T. McSorley , D. C. McMillan , P. G. Horgan , and D. Mansouri , “The Relationship Between Systemic Inflammatory Response, Screen Detection and Outcome in Colorectal Cancer,” Colorectal Disease 26 (2024): 81–94, 10.1111/codi.16824. 38095280

[15] Y. Yin , Y. Zhang , L. Li , S. Zhang , N. Liu , and S. Yuan , “Prognostic Value of Pretreatment Lymphocyte‐to‐Monocyte Ratio and Development of a Nomogram in Breast Cancer Patients,” Frontiers in Oncology 11 (2021): 650980, 10.3389/fonc.2021.650980. 34976782 PMC8719671

[16] J. Zhou , S. Wei , X. Guo , et al., “Correlation Between Preoperative Peripheral Blood NLR, PLR, LMR and Prognosis of Patients With Head and Neck Squamous Cell Carcinoma,” BMC Cancer 23, no. 1 (2023): 1247, 10.1186/s12885-023-11752-y. 38110870 PMC10726578

[17] J. M. Josse , M. C. Cleghorn , K. M. Ramji , et al., “The Neutrophil‐to‐Lymphocyte Ratio Predicts Major Perioperative Complications in Patients Undergoing Colorectal Surgery,” Colorectal Disease: The Official Journal of the Association of Coloproctology of Great Britain and Ireland 18, no. 7 (2016): O236–O242, 10.1111/codi.13373. 27154050

[18] B. W. Shi , L. Xu , C. X. Gong , et al., “Preoperative Neutrophil to Lymphocyte Ratio Predicts Complications After Esophageal Resection That Can Be Used as Inclusion Criteria for Enhanced Recovery After Surgery,” Frontiers in Surgery 9 (2022): 897716, 10.3389/fsurg.2022.897716. 35910480 PMC9326077

[19] S. H. Park , J. K. Lim , M. K. Kang , et al., “Predictive Factors for Severe Radiation‐Induced Lung Injury in Patients With Lung Cancer and Coexisting Interstitial Lung Disease,” Radiotherapy and Oncology 192 (2024): 110053, 10.1016/j.radonc.2023.110053. 38104782

[20] D. Yang , P. Li , Z. Meng , et al., “Combined Pretreatment Neutrophil‐Lymphocyte Ratio and Platelet‐Lymphocyte Ratio Predicts Survival and Prognosis in Patients With Non‐Metastatic Nasopharyngeal Carcinoma: A Retrospective Study,” Scientific Reports 14, no. 1 (2024): 9898, 10.1038/s41598-024-59131-2. 38688967 PMC11061272

[21] Scottish Government , “Scottish Index of Multiple Deprivation—gov.scot 2020,” accessed September 16, 2024, https://www.gov.scot/collections/scottish‐index‐of‐multiple‐deprivation‐2020/.

[22] S. Subramaniam , J. J. Aalberg , R. P. Soriano , and C. M. Divino , “New 5‐Factor Modified Frailty Index Using American College of Surgeons NSQIP Data,” Journal of the American College of Surgeons 226 (2018): 173–181, 10.1016/j.jamcollsurg.2017.11.005. 29155268

[23] J. McGovern , A. Grayston , D. Coates , et al., “The Relationship Between the Modified Frailty Index Score (mFI‐5), Malnutrition, Body Composition, Systemic Inflammation and Short‐Term Clinical Outcomes in Patients Undergoing Surgery for Colorectal Cancer,” BMC Geriatrics 23, no. 1 (2023): 9, 10.1186/s12877-022-03703-2. 36609242 PMC9817261

[24] “RStudio: Integrated Development for R,” 2020.

[25] E. V. King , C. H. Ottensmeier , and G. J. Thomas , “The Immune Response in HPV + Oropharyngeal Cancer,” Oncoimmunology 3 (2014): e27254, 10.4161/onci.27254. 24575385 PMC3916354

[26] P. De Luca , A. Di Stadio , G. Petruzzi , et al., “A Multicenter Protocol to Assess the Prognostic Significance of the Tumor Microenvironment in Patients With Squamous Cell Carcinoma of the Larynx,” Head & Neck 46 (2024): 2123–2131, 10.1002/hed.27860. 38958199

[27] Y. Zhu , M. Li , C. Bo , et al., “Prognostic Significance of the Lymphocyte‐to‐Monocyte Ratio and the Tumor‐Infiltrating Lymphocyte to Tumor‐Associated Macrophage Ratio in Patients With Stage T3N0M0 Esophageal Squamous Cell Carcinoma,” Cancer Immunology, Immunotherapy 66 (2017): 343–354, 10.1007/s00262-016-1931-5. 27915370 PMC11029213

[28] F. Milana , M. A. Polidoro , C. Soldani , et al., “Unveiling the Prognostic Role of Blood Inflammatory Indexes in a Retrospective Cohort of Patients Undergoing Liver Resection for Intrahepatic Cholangiocarcinoma,” International Journal of Surgery 110 (2024): 7088–7096, 10.1097/JS9.0000000000001924. 38967502 PMC11573080

[29] X. Hu , T. Tian , Q. Sun , and W. Jiang , “Prognostic Value of the Neutrophil‐to‐Lymphocyte Ratio and Platelet‐to‐Lymphocyte Ratio in Laryngeal Cancer: What Should We Expect From a Meta‐Analysis?,” Frontiers in Oncology 12 (2022): 945820, 10.3389/fonc.2022.945820. 36033468 PMC9400104

[30] F. Yang , Q. Huang , Z. Guan , and Q. Diao , “Prognostic Significance of Pretreatment Neutrophil‐to‐Lymphocyte Ratio in Patients With Laryngeal Cancer: A Systematic Review and Meta‐Analysis,” European Archives of Oto‐Rhino‐Laryngology 278 (2021): 417–425, 10.1007/s00405-020-06337-5. 32886184

[31] M. Kara , S. Uysal , U. Altinişik , S. Cevizci , O. Güçlü , and F. S. Dereköy , “The Pre‐Treatment Neutrophil‐To‐Lymphocyte Ratio, Platelet‐to‐Lymphocyte Ratio, and Red Cell Distribution Width Predict Prognosis in Patients With Laryngeal Carcinoma,” European Archives of Oto‐Rhino‐Laryngology 274 (2017): 535–542, 10.1007/s00405-016-4250-8. 27520567

[32] L. Bras , D. A. J. J. Driessen , J. de Vries , et al., “Patients With Head and Neck Cancer: Are They Frailer Than Patients With Other Solid Malignancies?,” European Journal of Cancer Care (England) 29 (2020): e13170, 10.1111/ecc.13170. PMC7063690 31571340

[33] P. Dewansingh , L. Bras , L. ter Beek , et al., “Malnutrition Risk and Frailty in Head and Neck Cancer Patients: Coexistent but Distinct Conditions,” European Archives of Oto‐Rhino‐Laryngology 280 (2023): 1893–1902, 10.1007/s00405-022-07728-6. 36484854 PMC9988738

[34] C. L. Nieman , K. T. Pitman , A. P. Tufaro , D. W. Eisele , K. D. Frick , and C. G. Gourin , “The Effect of Frailty on Short‐Term Outcomes After Head and Neck Cancer Surgery,” Laryngoscope 128 (2018): 102–110, 10.1002/lary.26735. 28731497

[35] W. Xu , Y. Liang , and Z. Lin , “Association Between Neutrophil–Lymphocyte Ratio and Frailty: The Chinese Longitudinal Healthy Longevity Survey,” Frontiers in Medicine (Lausanne) 8 (2021): 783077, 10.3389/fmed.2021.783077. PMC8761893 35047530

[36] P. Gaudioso , D. Borsetto , J. Polesel , et al., “Blood Markers Predicting Clinically Occult Lymph Node Metastasis in Head and Neck Squamous Cell Carcinoma,” Operations Research Letters 86 (2024): 32–40, 10.1159/000534079. 37972574

[37] D. D. Sharbel , M. Abkemeier , M. W. Groves , W. G. Albergotti , J. K. Byrd , and C. Reyes‐Gelves , “Occult Metastasis in Laryngeal Squamous Cell Carcinoma: A Systematic Review and Meta‐Analysis,” Annals of Otology, Rhinology and Laryngology 130 (2021): 67–77, 10.1177/0003489420937744. 32608245

[38] O. Gallo , A. Deganello , J. Scala , and E. De Campora , “Laryngeal Cancer • N0 Neck • Neck Dissection Cancro Della Laringe • N0 • Svuotamento Laterocervicale Evolution of Elective Neck Dissection in N0 Laryngeal Cancer Evoluzione Dello Svuotamento Elettivo Del Collo Nel Carcinoma Laringeo N0,” vol. 26. 2006. PMC2639990 17633152

[39] E. Bocca , C. Calearo , V. I. De , T. Marullo , G. Motta , and A. Ottaviani , “Occult Metastases in Cancer of the Larynx and Their Relationship to Clinical and Histological Aspects of the Primary Tumor: A Four‐Year Multicentric Research,” Laryngoscope 94 (1984): 1086–1090, 10.1288/00005537-198408000-00017. 6748835

[40] P. A. Walker , B. Kunjuraman , and D. C. C. Bartolo , “Neutrophil‐To‐Lymphocyte Ratio Predicts Anastomotic Dehiscence,” ANZ Journal of Surgery 88 (2018): E573–7, 10.1111/ans.14369. 29377500

[41] Y. Tian , R. Li , G. Wang , K. Xu , H. Li , and L. He , “Prediction of Postoperative Infectious Complications in Elderly Patients With Colorectal Cancer: A Study Based on Improved Machine Learning,” BMC Medical Informatics and Decision Making 24, no. 1 (2024): 11, 10.1186/s12911-023-02411-0. 38184556 PMC10770876

[42] Y. Heo , H. S. Lee , S. Jung , et al., “Improved Early Detection Models of Pharyngocutaneous Fistula After Total Laryngectomy,” Journal of Clinical Medicine 12, no. 5 (2023): 1851, 10.3390/jcm12051851. 36902638 PMC10003396

[43] S. Matsumoto , M. Nakayama , M. Gosho , et al., “Inflammation‐Based Score (Combination of Platelet Count and Neutrophil‐to‐Lymphocyte Ratio) Predicts Pharyngocutaneous Fistula After Total Laryngectomy,” Laryngoscope 132, no. 8 (2022): 1582–1587, 10.1002/lary.29970. 34870336

[44] N. Cichowska‐Cwalińska , M. Bieńkowski , M. Popęda , et al., “Radiotherapy‐Induced Dynamic Changes in the Lymphocyte‐to‐Monocyte Ratio in Patients With Laryngeal Cancer Indicate Poor Prognosis,” Frontiers in Oncology 13 (2023): 1234953, 10.3389/fonc.2023.1234953. 37886164 PMC10598385

[45] B. Bojaxhiu , A. J. Templeton , O. Elicin , et al., “Relation of Baseline Neutrophil‐to‐Lymphocyte Ratio to Survival and Toxicity in Head and Neck Cancer Patients Treated With (Chemo‐) Radiation,” Radiation Oncology (London, England) 13, no. 1 (2018): 216, 10.1186/s13014-018-1159-y. 30400969 PMC6219022

[46] J. N. Mandrekar , “Receiver Operating Characteristic Curve in Diagnostic Test Assessment,” Journal of Thoracic Oncology 5 (2010): 1315–1316, 10.1097/JTO.0b013e3181ec173d. 20736804

[47] N. Woodley , A. D. G. Rogers , K. Turnbull , et al., “Prognostic Scores in Laryngeal Cancer,” European Archives of Oto‐Rhino‐Laryngology 279 (2022): 3705–3715, 10.1007/s00405-021-07233-2. 35112153

[48] T. Kawano , T. Hirano , K. Tateyama , K. Yoshinaga , K. Shinomura , and M. Suzuki , “Prognostic Value of Pretreatment Inflammatory Biomarkers in Patients With Laryngeal Cancer,” Asian Journal of Surgery 47 (2024): 2144–2151, 10.1016/j.asjsur.2024.01.073. 38311505

[49] H. Chen , S. Song , L. Zhang , W. Dong , X. Chen , and H. Zhou , “Preoperative Platelet–Lymphocyte Ratio Predicts Recurrence of Laryngeal Squamous Cell Carcinoma,” Future Oncology 16 (2020): 209–217, 10.2217/fon-2019-0527. 31984780

