# Correction to “Pre‐Treatment Serum Prognostic Scores and Survival in Curatively Treated Laryngeal Cancer”

**DOI:** 10.1002/lio2.70438

**Published:** 2026-05-11

R. Hurley , J. Osbourne , G. J. Inman , et al., “Pre‐Treatment Serum Prognostic Scores and Survival in Curatively Treated Laryngeal Cancer,” Laryngoscope Investigative Otolaryngology 10, no. 2 (2025): e70124, 10.1002/lio2.70124.40124253 PMC11929121

There are transcriptional errors in the Results section under Section 3.2 that the authors wish to correct. These relate to the reported numbers of patients classified into high and low prognostic score groups, and to the reported threshold for LMR. The correct values were used in the subsequent analyses; therefore, these corrections do not alter the analysis, results, or conclusions of the article.

3.2 (a) NLR

The text currently reads:

“For OS, the NLR threshold was 2.34, to give a sensitivity of 0.72 and specificity of 0.47, and an AUC of 0.62. Two hundred and ninety‐six patients had high NLR, and 179 had low NLR by this threshold. For CSS, the NLR threshold was 2.98, with sensitivity of 0.58 and specificity of 0.62, with an AUC of 0.62. Two hundred and seven patients had high NLR, and 267 had low NLR by this threshold.”

This should read:

“For OS, the NLR threshold was 2.34, to give a sensitivity of 0.72 and specificity of 0.47, and an AUC of 0.62. Two hundred and ninety‐five patients had high NLR, and 178 had low NLR by this threshold. For CSS, the NLR threshold was 2.98, with sensitivity of 0.58 and specificity of 0.62, with an AUC of 0.62. Two hundred and six patients had high NLR, and 267 had low NLR by this threshold.”

(b) PLR

The text currently reads:

“For OS, the PLR threshold was 142.5, with a sensitivity of 0.58 and specificity of 0.59, with an AUC of 0.57. By this threshold, 231 patients had high PLR and 243 had low PLR. For CSS, the PLR threshold was 152.1, with a sensitivity of 0.52 and a specificity of 0.62, with an AUC of 0.59; 191 patients had high PLR and 283 had low PLR by this threshold. For RFS, the threshold was 156.2, with a sensitivity of 0.47, a specificity of 0.65, and an AUC of 0.56. By this threshold, 181 patients had high PLR, and 292 had low PLR.”

This should read:

“For OS, the PLR threshold was 142.5, with a sensitivity of 0.58 and specificity of 0.59, with an AUC of 0.57. By this threshold, 231 patients had high PLR and 242 had low PLR. For CSS, the PLR threshold was 152.1, with a sensitivity of 0.52 and a specificity of 0.62, with an AUC of 0.59; 191 patients had high PLR and 282 had low PLR by this threshold. For RFS, the threshold was 156.2, with a sensitivity of 0.47, a specificity of 0.65, and an AUC of 0.56. By this threshold, 181 patients had high PLR, and 292 had low PLR.”

(c) LMR

The text currently reads:

“For OS, the LMR threshold was 2.33, with a sensitivity of 0.44 and specificity of 0.78, with an AUC of 0.63. For CSS, the LMR threshold was also 2.33, with a sensitivity of 0.49 and specificity of 0.73, with an AUC of 0.64. Three hundred and eighteen patients had high LMR and 157 had low LMR by this threshold. For RFS, the LMR threshold was 2.21, with a sensitivity of 0.42 and specificity of 0.72, with an AUC 0.55. By this threshold, 310 patients had high LMR and 162 had low LMR.”

This should read:

“For OS, the LMR threshold was 2.23, with a sensitivity of 0.44 and specificity of 0.78, with an AUC of 0.63. For CSS, the LMR threshold was also 2.23, with a sensitivity of 0.49 and specificity of 0.73, with an AUC of 0.64. Three hundred and eighteen patients had high LMR and 155 had low LMR by this threshold. For RFS, the LMR threshold was 2.21, with a sensitivity of 0.42 and specificity of 0.72, with an AUC 0.55. By this threshold, 322 patients had high LMR and 151 had low LMR.”

(d) SIII

The text currently reads:

“For OS, the SIII threshold was 675.12, with a sensitivity of 0.59 and a specificity of 0.57, with an AUC of 0.59. For CSS, the SIII threshold was also 675.12, with a sensitivity of 0.65 and a specificity of 0.55, with an AUC of 0.61. Two hundred and forty patients had high SIII and 233 had low SIII. For RFS, the SIII threshold was 969.7, with a sensitivity of 0.44 and a specificity of 0.7, with an AUC of 0.57. By this threshold, 311 patients had high SIII, and 161 had low SIII.”

This should read:

“For OS, the SIII threshold was 675.12, with a sensitivity of 0.59 and a specificity of 0.57, with an AUC of 0.59. For CSS, the SIII threshold was also 675.12, with a sensitivity of 0.65 and a specificity of 0.55, with an AUC of 0.61. Two hundred and forty patients had high SIII and 233 had low SIII. For RFS, the SIII threshold was 969.7, with a sensitivity of 0.44 and a specificity of 0.7, with an AUC of 0.57. By this threshold, 162 patients had high SIII, and 311 had low SIII.”

Due to the errors noted, Table S2 has also been revised online.

We apologize for these errors.

## Supporting information


**Table S1:** Method of calculation of each prognostic score.
**Table S2:** Sensitivity, specificity, AUC and univariate survival analysis for OS, CSS, RFS for NLR, PLR, LMR and SIII.
**Table S3:** Different clinical characteristics of groups based on prognostic score cutoffs.
**Figure S1:** Kaplan Meier curves demonstrating univariate survival analysis for OS, CSS and RFS for NLR, PLR, LMR, SIII and risk groups (*N* = 473). All were statistically significant in OS/CSS/RFS. *p*‐values were calculated using log‐rank testing.
**Figure S2:** Kaplan Meier curves for univariate analysis of sex, smoking status, site, stage, nodal status, deprivation, frailty, performance status and treatment with regards to OS, CSS, RFS. Age was analysed as a continuous variable using a cox proportional hazards model (See methods text). For OS—age, site, stage, smoking, PS, frailty and nodal status were found to be significant predictors on univariate analysis. For CSS, site, stage, PS and nodal status were found to be significant predictors. For RFS, nodal status, stage and PS was found to be significant. Significant predictors on univariate analysis were included in multivariate analysis. *p*‐values were calculated using log‐rank testing.
**Table S4:** Multivariate survival analysis by prognostic score.

