# Supplementary material for: Pre‐Treatment Serum Prognostic Scores and Survival in Curatively Treated Laryngeal Cancer
Source: Laryngoscope Investig Otolaryngol. 2025 Mar 22;10(2):e70124. doi: 10.1002/lio2.70124 (PMC11929121; doi:10.1002/lio2.70124)
Supplement: Supplementary file 1 — Data S1. Supporting Information. [file LIO2-10-e70124-s001.docx]

SUPPLEMENTARY FIGURES/TABLES

| Supplementary Table 1. Method of calculation of each prognostic score | |
| --- | --- |
| Serum prognostic marker | **Calculation method** |
| Neutrophil-to-lymphocyte ratio (NLR) | neutrophils/lymphocytes |
| Platelet-to-lymphocyte ratio (PLR) | platelets/lymphocytes |
| Lymphocyte-to-monocyte ratio (LMR) | lymphocytes/monocytes |
| Systemic-immune-inflammation index (SIII) | platelets * neutrophils/lymphocytes |

| Supplementary Table 2. Sensitivity, specificity, AUC and univariate survival analysis for OS, CSS, RFS for NLR, PLR, LMR and SIII | | | | |
| --- | --- | --- | --- | --- |
| Score | NLR | PLR | LMR | SIII |
| Parameter |  |  |  |  |
| OS threshold  *No of patients above*  *No of patients below* | 2.34  296  179 | 142.5  231  243 | 2.33  318  157 | 675.12  240  233 |
| OS sensitivity | 0.72 | 0.58 | 0.44 | 0.59 |
| OS specificity | 0.47 | 0.59 | 0.78 | 0.57 |
| OS AUC | 0.62 | 0.57 | 0.63 | 0.59 |
| OS univariate survival analysis p value | **<0.0001** | **0.0052** | **<0.0001** | **0.00097** |
| CSS threshold  *No of patients above*  *No of patients below* | 2.98  207  268 | 152.1  191  283 | 2.33  318  157 | 675.12  240  233 |
| CSS sensitivity | 0.58 | 0.52 | 0.49 | 0.65 |
| CSS specificity | 0.62 | 0.62 | 0.73 | 0.55 |
| CSS AUC | 0.62 | 0.59 | 0.64 | 0.61 |
| CSS univariate survival analysis p value | **<0.0001** | **0.00078** | **<0.0001** | **0.00021** |
| RFS threshold  *No of patients above*  *No of patients below* | 3.63  151  322 | 156.2  181  292 | 2.21  310  162 | 969.7  162  311 |
| RFS sensitivity | 0.4 | 0.47 | 0.42 | 0.44 |
| RFS specificity | 0.71 | 0.65 | 0.72 | 0.7 |
| RFS AUC | 0.56 | 0.56 | 0.55 | 0.57 |
| RFS univariate survival analysis p value | **0.00081** | **0.0017** | **0.00016** | **0.00059** |

| **Supplementary Table 3. Different clinical characteristics of groups based on prognostic score cutoffs** | | | | | | | | | | | | |
| --- | --- | --- | --- | --- | --- | --- | --- | --- | --- | --- | --- | --- |
| **Characteristic**  *Patient n=473* | **High NLR** | **Low NLR** | **pvalue** | **High PLR** | **Low PLR** | **pvalue** | **High LMR** | **Low LMR** | **pvalue** | **High SIII** | **Low SIII** | **pvalue** |
| **Mean age** | 63.9 | 64.1 | 0.76 | 64.2 | 63.7 | 0.33 | 63.1 | 65.6 | **0.02*** | 63.0 | 64.9 | 0.14 |
| **Gender**  Male  Female | 235  60 | 143  35 | 0.95 | 180  51 | 198  44 | 0.35 | 240  77 | 138  18 | **0.002*** | 188  52 | 190  43 | 0.449 |
| **Smoking**  Yes  No  Ex | 168  24  103 | 105  17  56 | 0.73 | 128  26  77 | 145  15  82 | 0.14 | 192  28  97 | 81  13  62 | 0.14 | 142  19  79 | 131  22  80 | 0.75 |
| **Stage**  Early  Advanced | 121  174 | 99  79 | **0.003*** | 99  132 | 121  121 | 0.14 | 159  158 | 61  95 | **0.03*** | 92  148 | 128  105 | **0.0004***** |
| **Subsite**  Glottic  Supraglottic  Subglottic  Transglottic  Indeterminate | 104  149  3  36  3 | 78  82  0  18  0 | 0.15 | 84  110  2  33  2 | 98  121  1  21  1 | 0.32 | 127  158  1  29  2 | 55  73  2  25  1 | 0.15 | 79  120  3  36  2 | 103  111  0  18  1 | **0.01*** |
| **Nodal status**  N0  N1  N2  N3 | 228  16  44  7 | 155  9  11  3 | **0.03*** | 180  13  32  6 | 203  12  23  4 | 0.39 | 260  15  35  7 | 123  10  20  3 | 0.80 | 176  16  41  7 | 207  9  14  3 | **0.0002***** |
| **Treatment**  RT only  Surgery only  Surgery and RT  ChemoRT  Trimodality | 124  107  42  15  7 | 81  69  15  9  4 | 0.46 | 98  86  30  10  7 | 107  90  27  14  4 | 0.76 | 139  120  36  16  6 | 66  56  21  8  5 | 0.86 | 93  93  33  13  8 | 112  83  24  11  3 | 0.19 |
| **Performance**  **Status**  0  1  2  3 | 137  113  41  4 | 90  72  16  0 | 0.16 | 105  89  33  4 | 122  96  24  0 | 0.08 | 164  120  32  1 | 63  65  25  3 | **0.02*** | 106  93  37  4 | 121  92  20  0 | **0.02*** |
| **Deprivation^**  1  2  3  4  5  Not found | 147  73  29  22  22  2 | 79  39  20  19  16  5 | 0.28 | 112  56  27  18  16  2 | 114  56  22  23  22  5 | 0.68 | 151  76  30  30  25  5 | 75  36  19  11  13  2 | 0.90 | 117  59  28  16  18  2 | 109  53  21  25  20  5 | 0.43 |
| **Frailty(by Mfi-5)**  Not frail  Moderately frail  Severely frail | 59  145  91 | 42  96  40 | 0.14 | 48  110  73 | 53  131  58 | 0.17 | 77  171  69 | 24  70  62 | **0.0001**** | 47  112  81 | 54  129  50 | **0.0157*** |

*Chi-square tests were used to assess differences between groups, except for mean age in which differences between groups were assessed using Mann Whitney-U test due to non-parametric distribution. ^Deprivation was measured by SIMD quintiles. P values <0.05 were defined as significant (*), with P values <0.001 highly significant (**).*


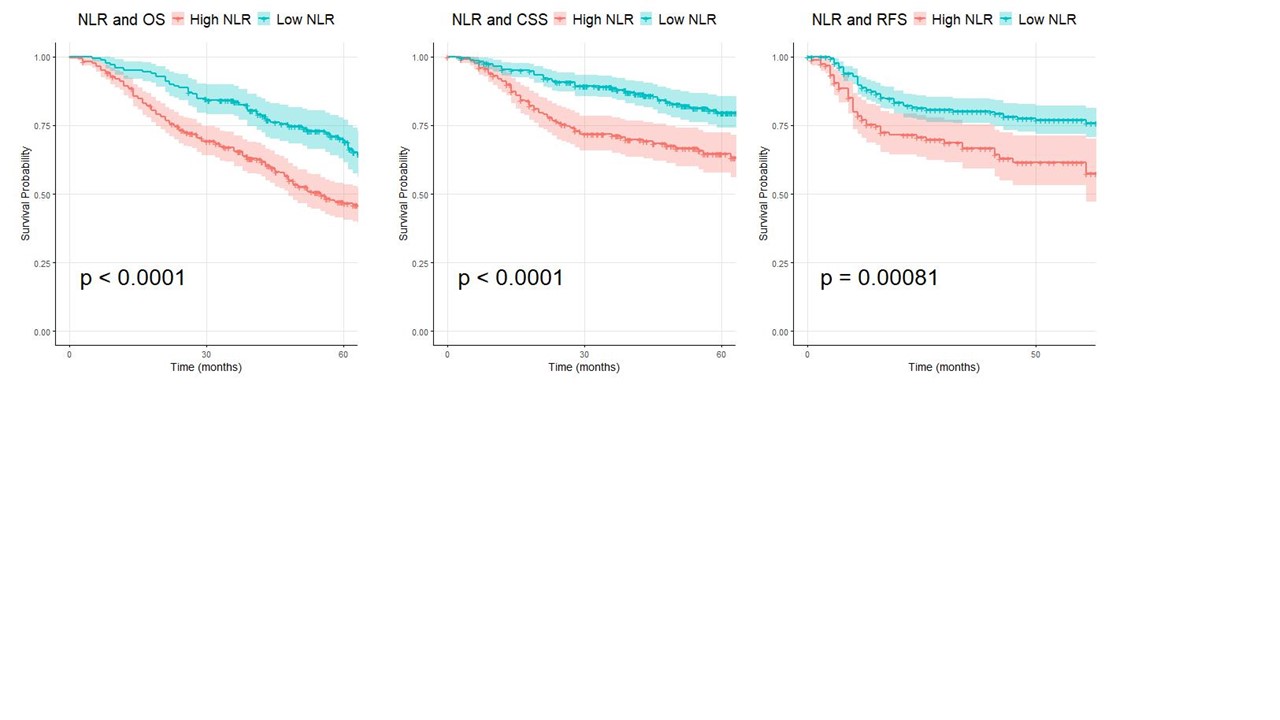


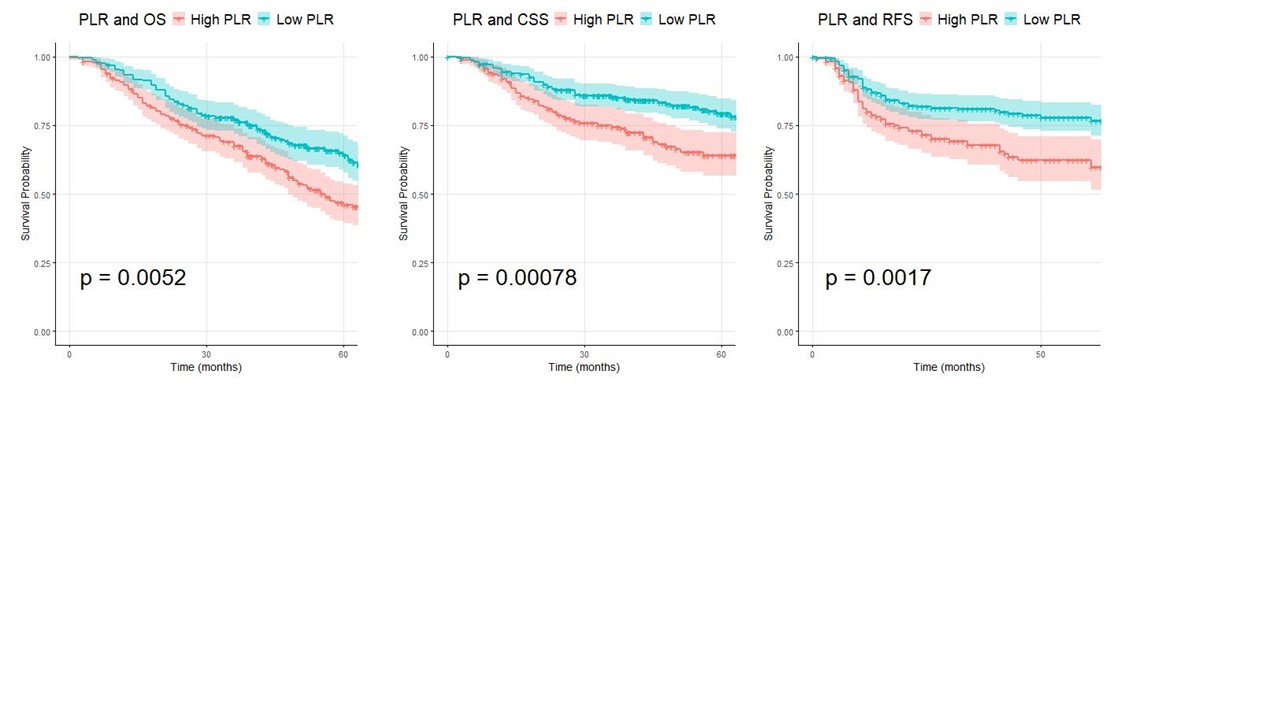


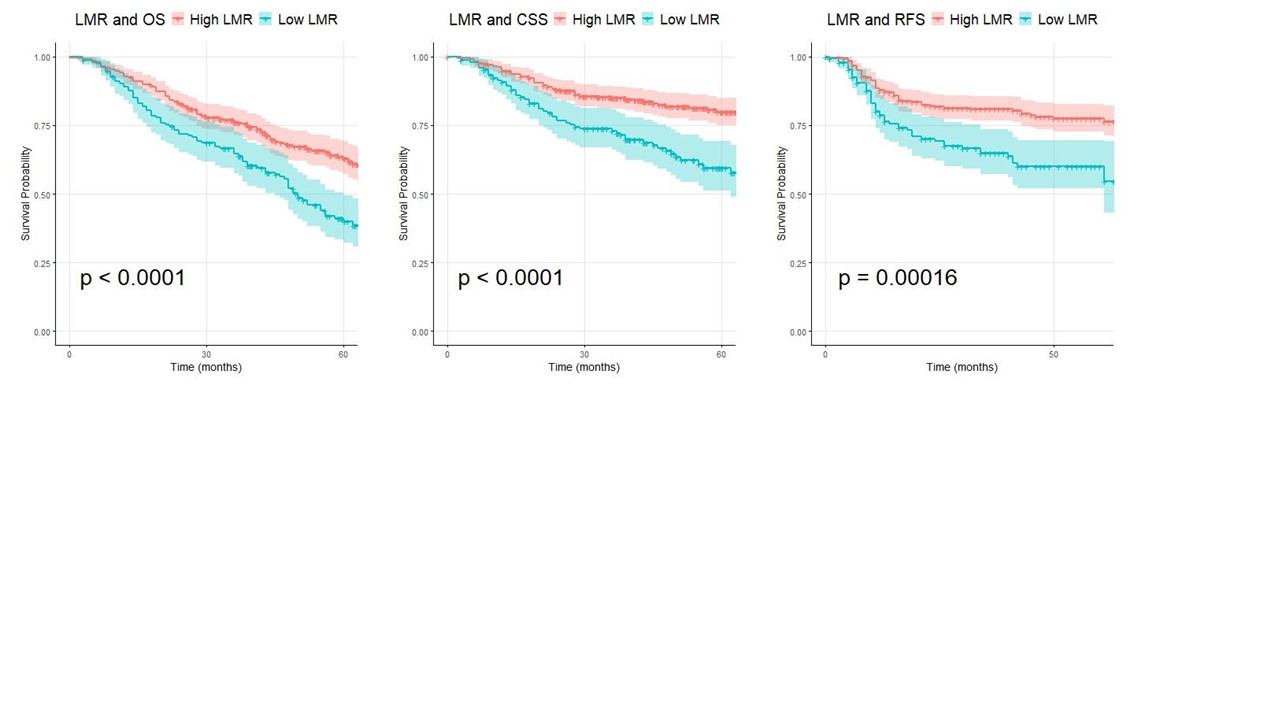


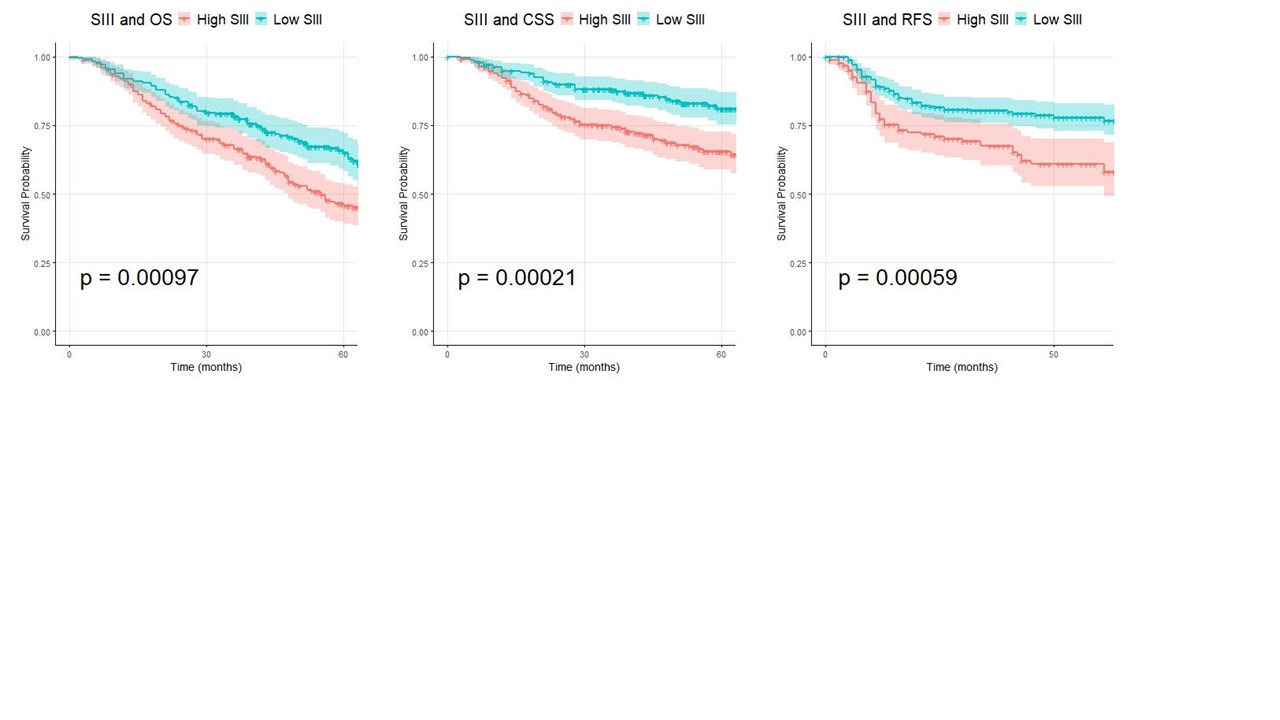


*
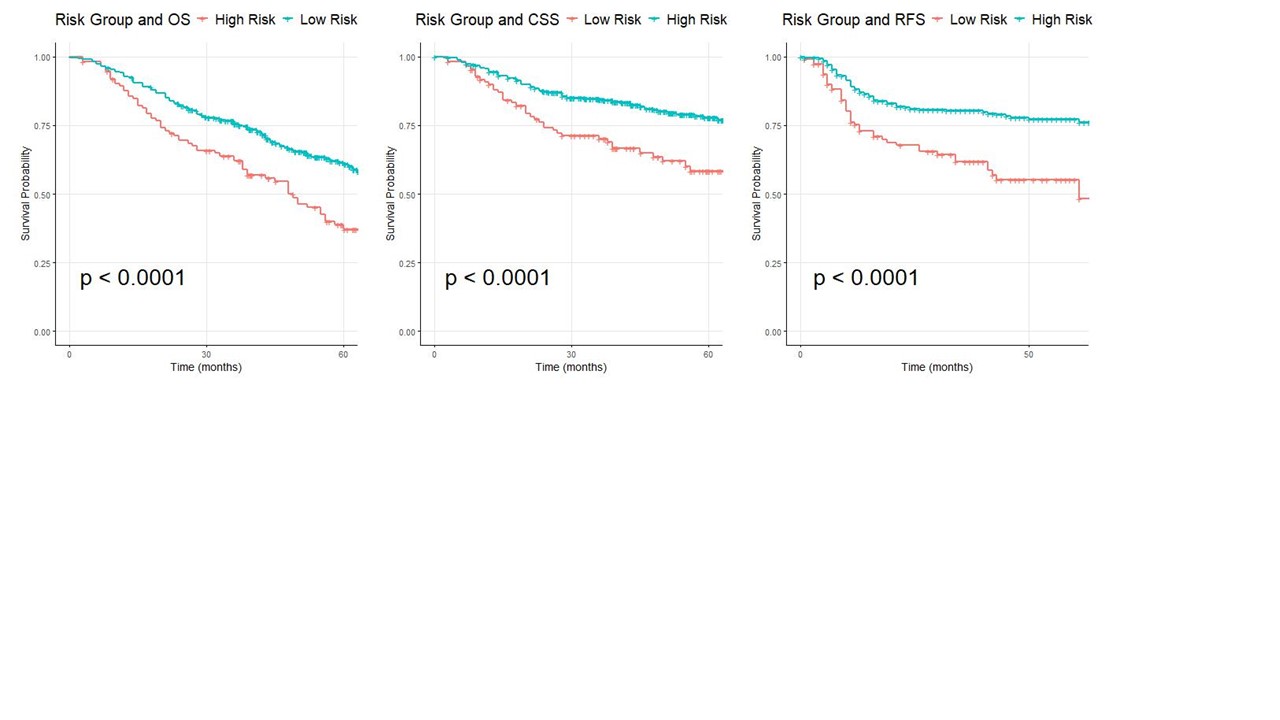
*

Supplementary Figure 1. Kaplan Meier curves demonstrating univariate survival analysis for OS, CSS and RFS for NLR, PLR, LMR, SIII and risk groups (N=473). All were statistically significant in OS/CSS/RFS. P-values were calculated using log-rank testing.


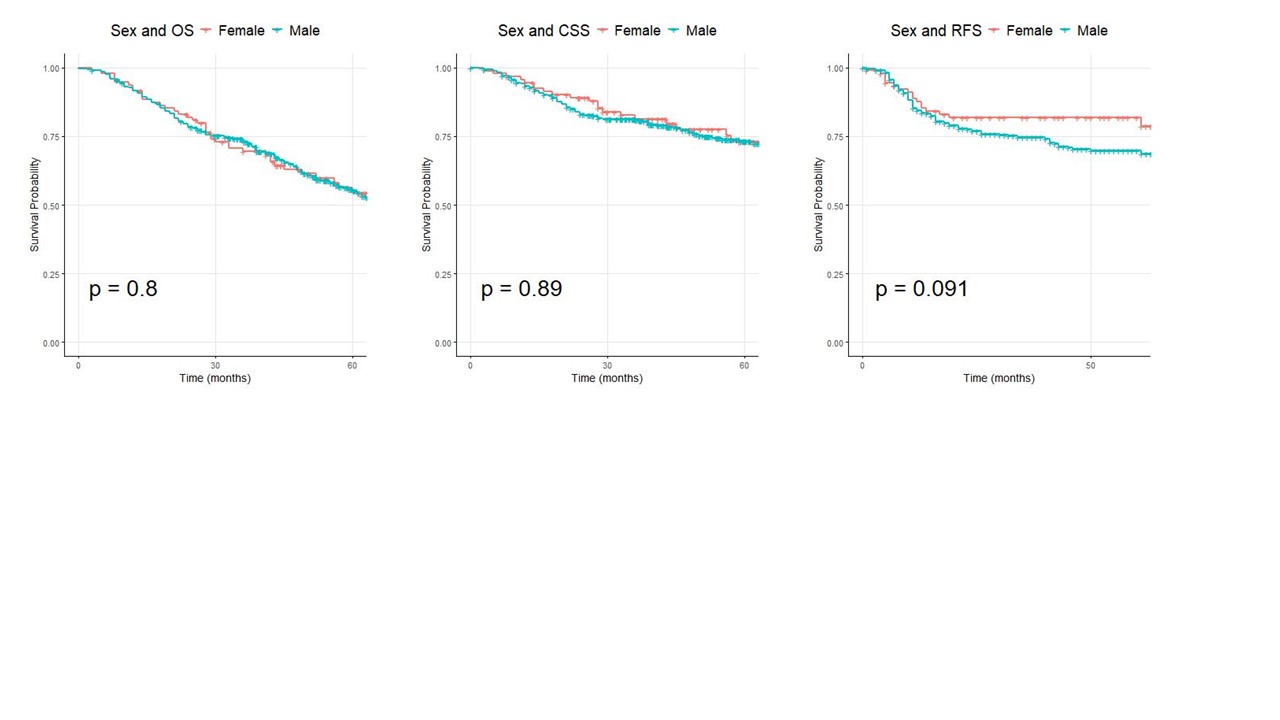


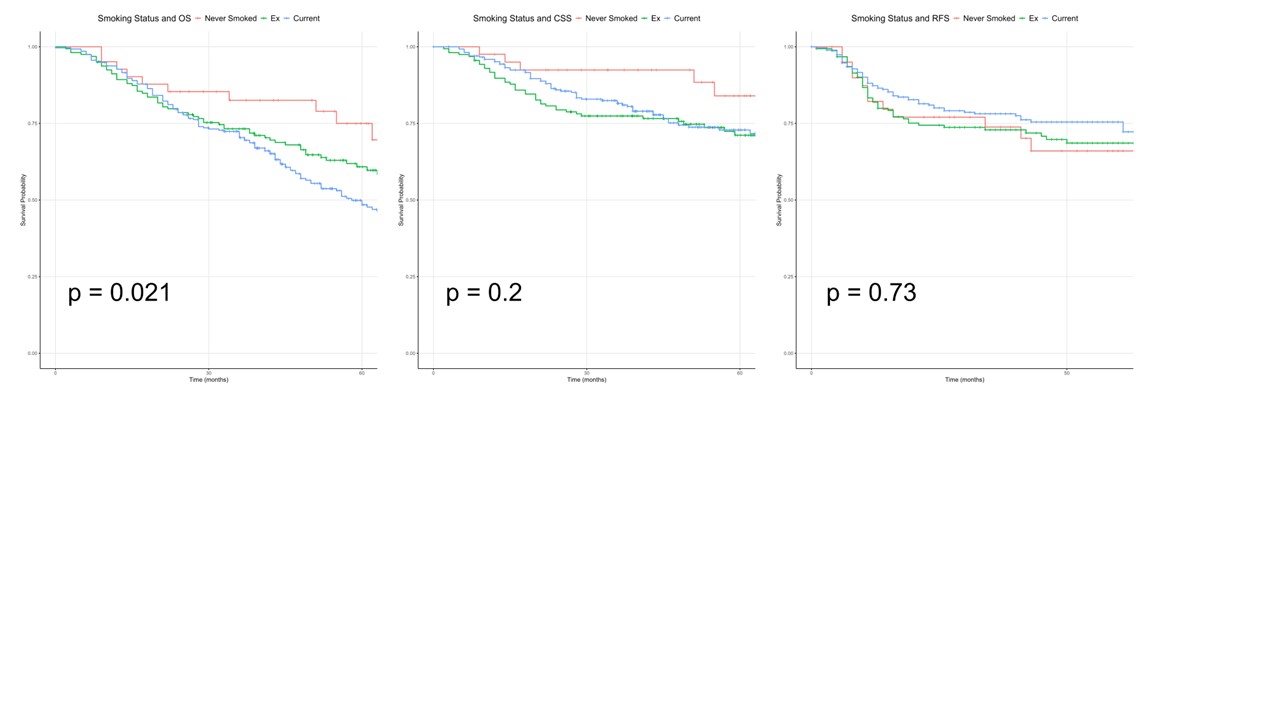


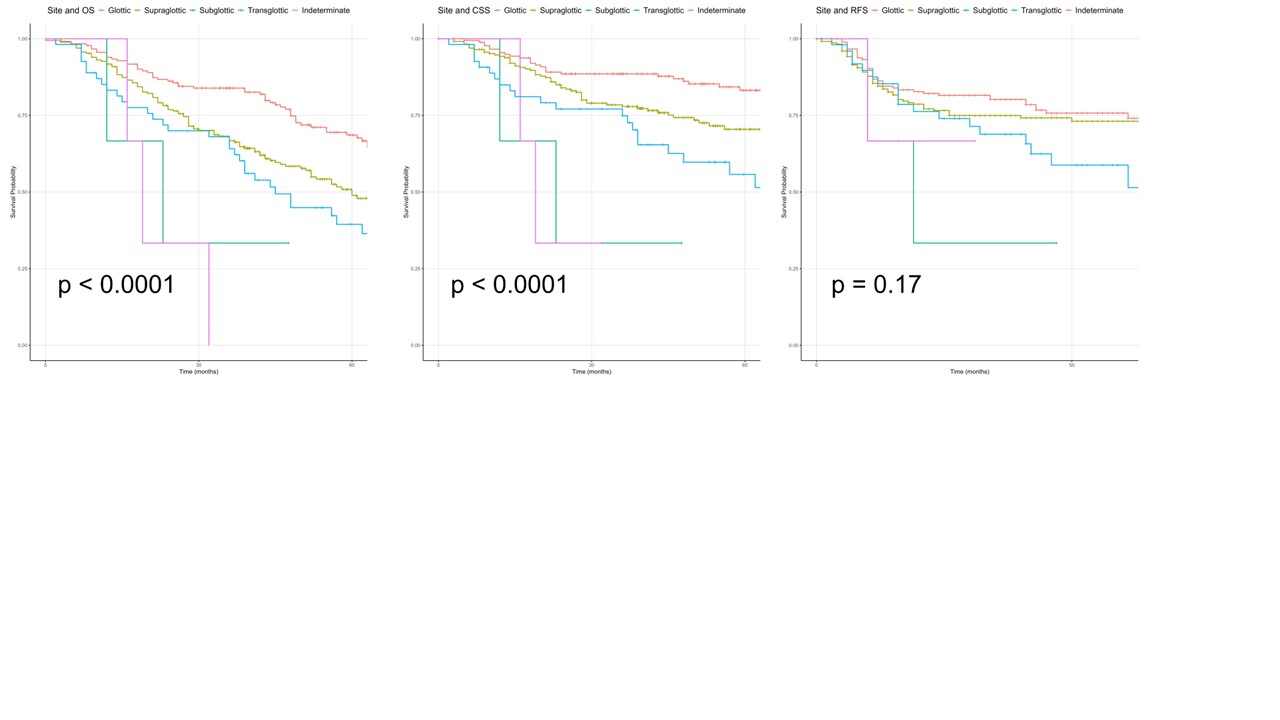


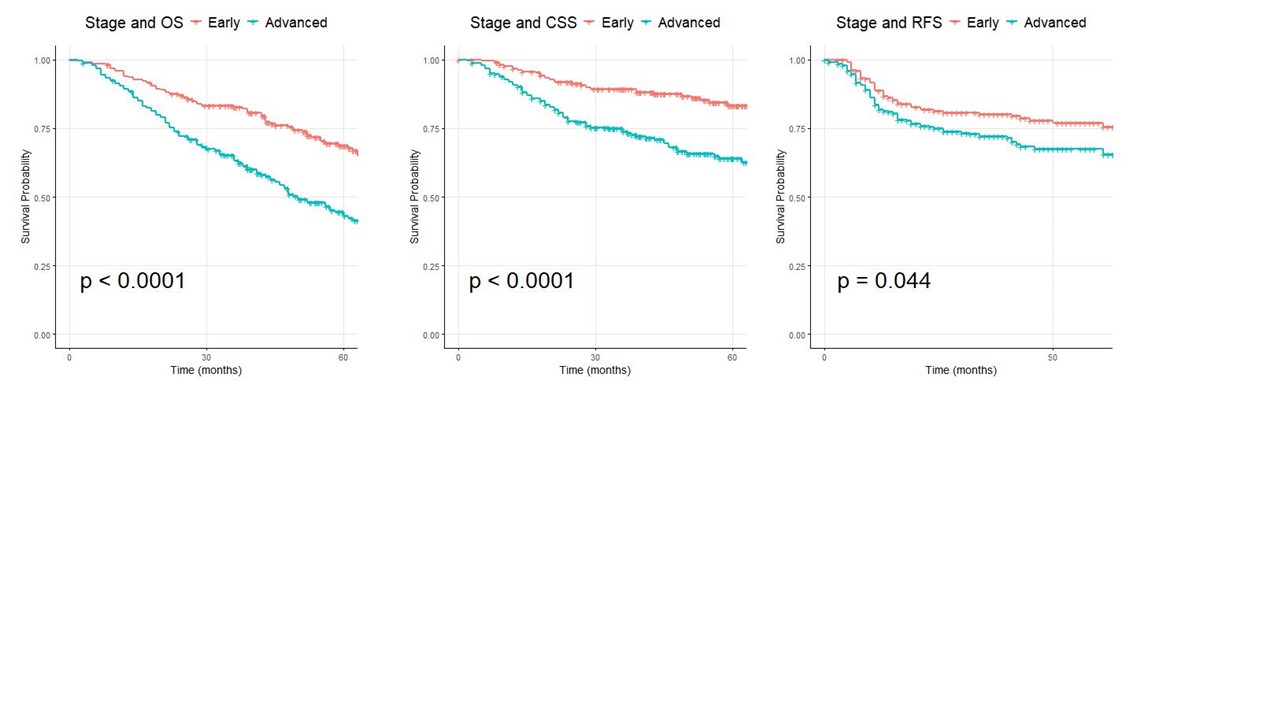


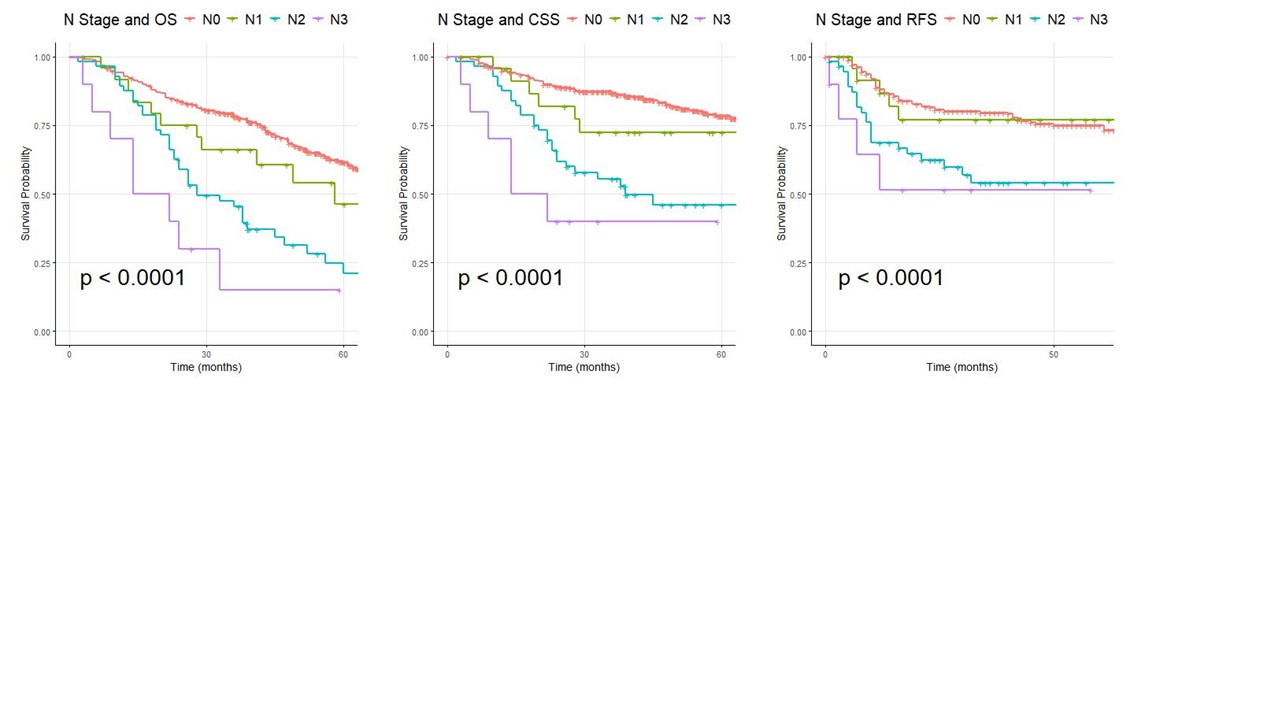


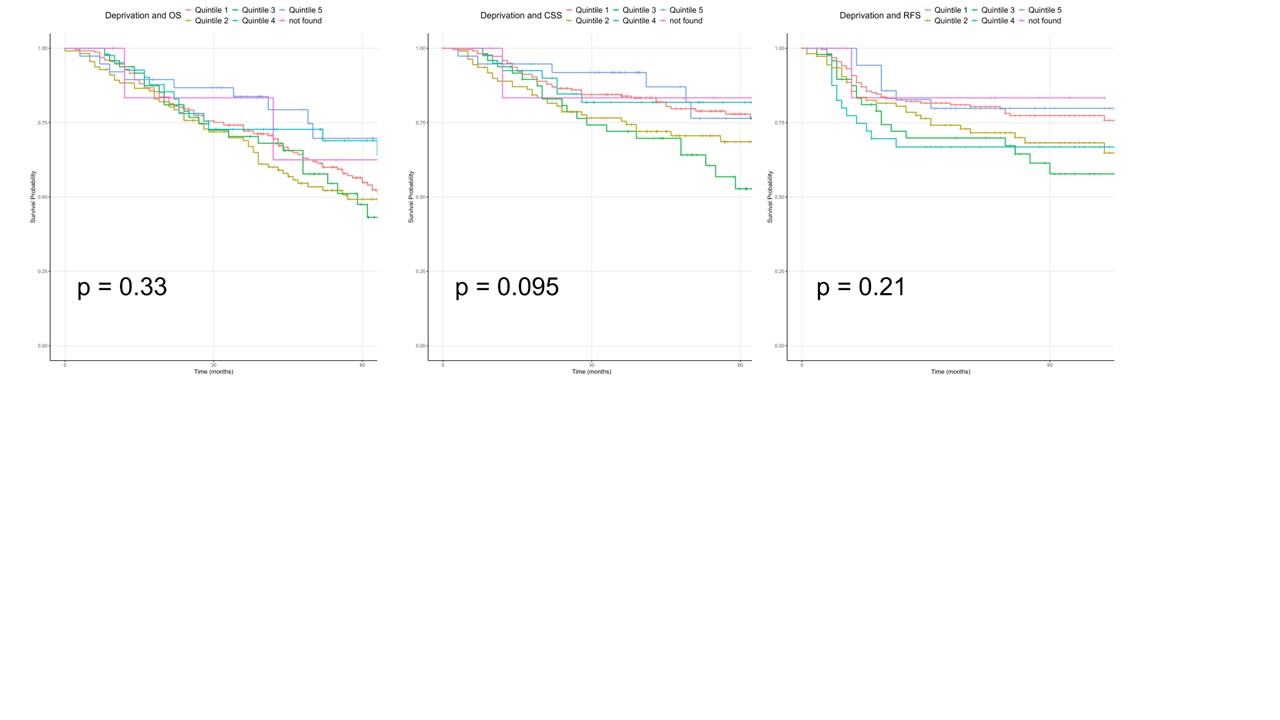


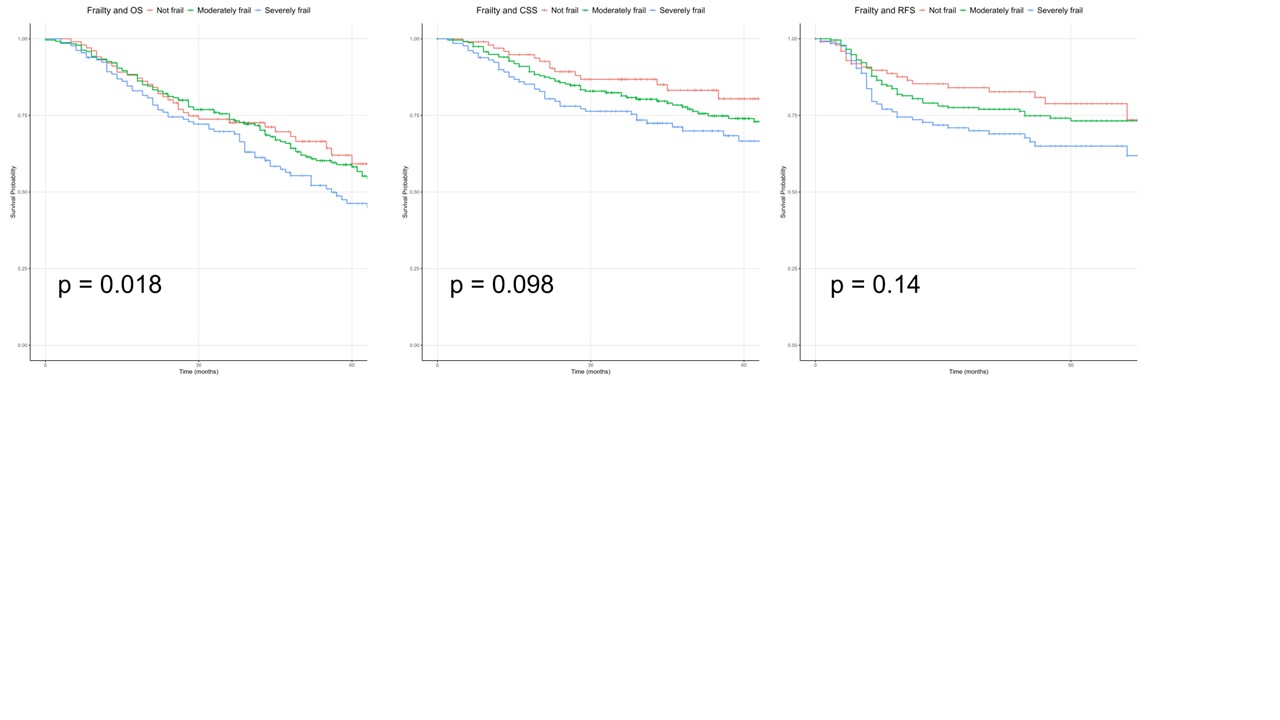


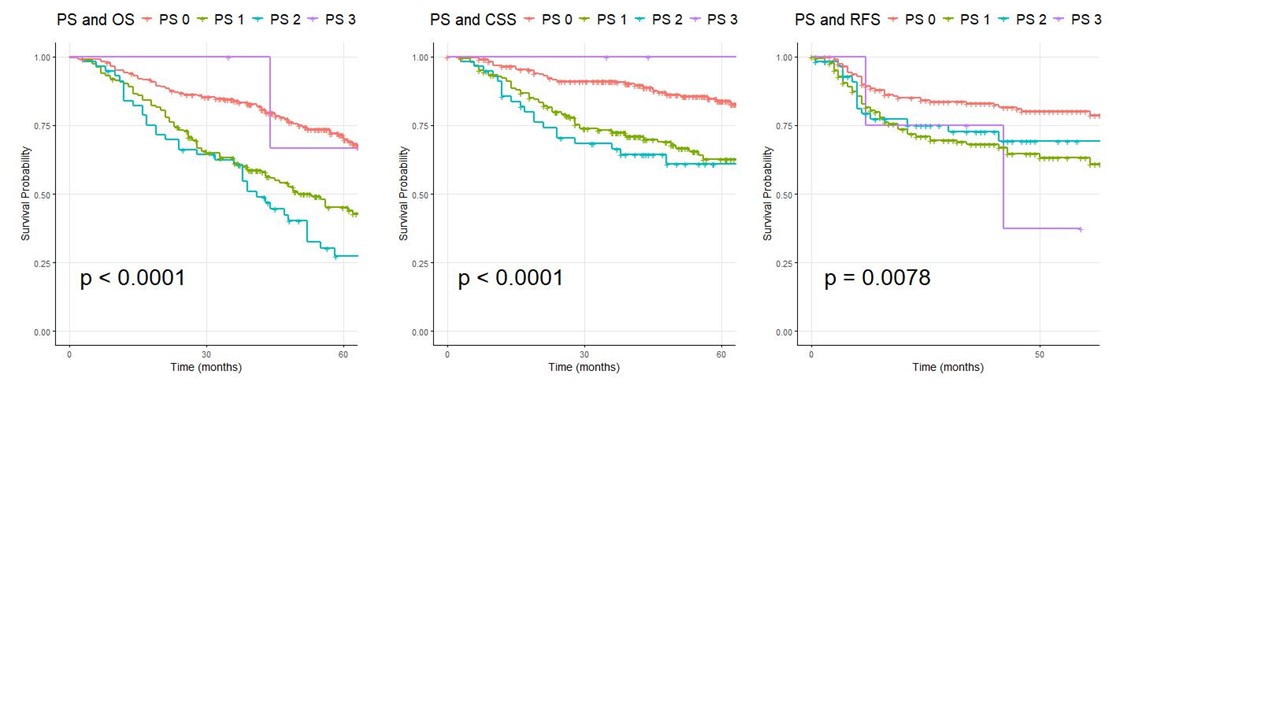


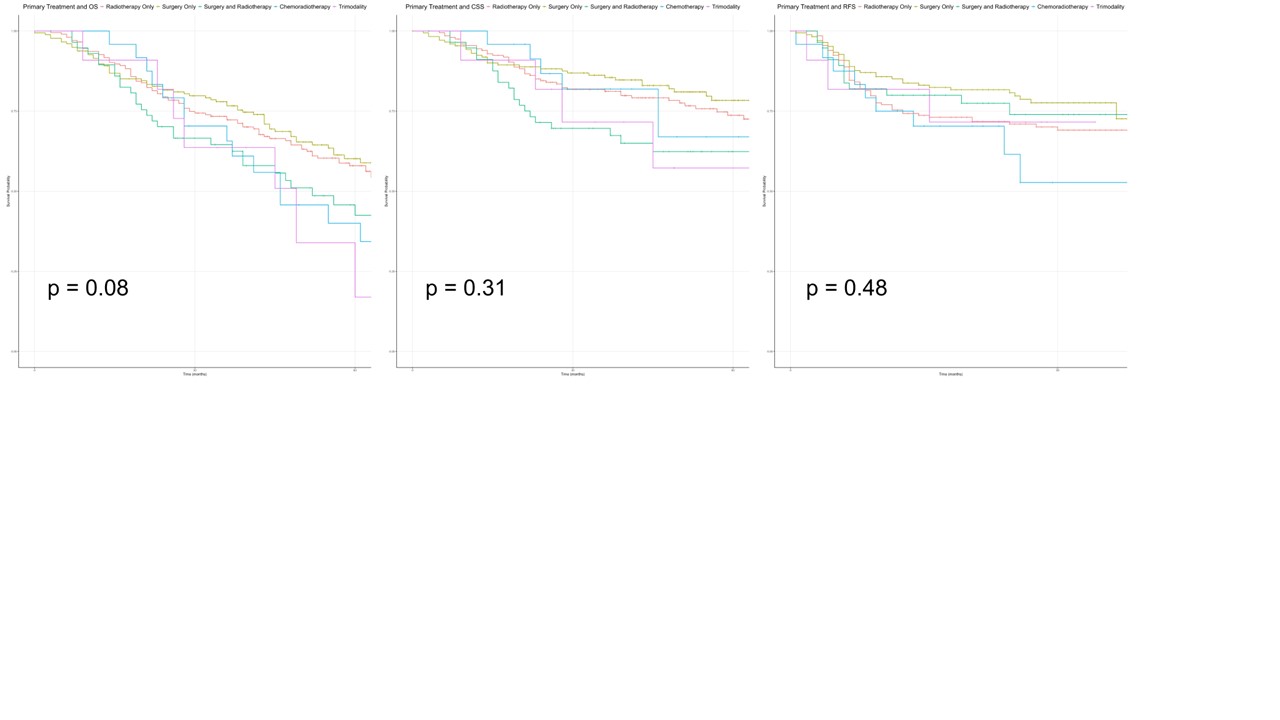


Supplementary Figure 2. Kaplan Meier curves for univariate analysis of sex, smoking status, site, stage, nodal status, deprivation, frailty, performance status and treatment with regards to OS, CSS, RFS. Age was analysed as a continuous variable using a cox proportional hazards model (See methods text). For OS – age, site, stage, smoking, PS, frailty and nodal status were found to be significant predictors on univariate analysis. For CSS, site, stage, PS and nodal status were found to be significant predictors. For RFS, nodal status, stage and PS was found to be significant. Significant predictors on univariate analysis were included in multivariate analysis. P-values were calculated using log-rank testing.

| **Supplementary Table 4. Multivariate Survival Analysis by Prognostic score** | | | | |
| --- | --- | --- | --- | --- |
| **Variable** | | **Hazard Ratio (HR)***^1^* | **95% CI***^1^* | **p-value** |
| **Overall Survival** | | | | |
| **Age** | | 1.03 | 1.01 - 1.05 | <0.001** |
| **Performance Status (Ref = 0)** | | - | - | - |
| *1* | | 1.68 | 1.23 - 2.28 | 0.001* |
| *2* | | 1.71 | 1.03- 2.82 | 0.036* |
| *3* | | 1.46 | 0.42 - 4.99 | 0.6 |
| **Smoking (Ref = Never Smoked)** | | - | - | - |
| *Ex* | | 1.27 | 0.69 - 2.35 | 0.4 |
| *Current* | | 1.84 | 1.00 - 3.37 | 0.049* |
| **Stage (Ref = Early)** | | - | - | - |
| *Advanced* | | 1.39 | 0.98 - 1.96 | 0.064 |
| **Subsite (Ref= Glottic)** | | - | - | - |
| *Supraglottic* | | 1.24 | 0.88 - 1.73 | 0.2 |
| *Subglottic* | | 2.70 | 0.62 - 11.8 | 0.2 |
| *Transglottic* | | 1.22 | 0.76 - 1.97 | 0.4 |
| *Indeterminate* | | 3.13 | 0.93 - 10.5 | 0.064 |
| **Nodal Status (Ref = N0)** | | - | - | - |
| *N1* | | 1.19 | 0.66 - 2.16 | 0.6 |
| *N2* | | 2.38 | 1.60 - 3.54 | <0.001** |
| *N3* | | 4.56 | 2.11 - 9.86 | <0.001** |
| **Frailty (Ref = Not frail)** | | - | - | - |
| *Moderately frail* | | 1.07 | 0.72 - 1.60 | 0.7 |
| *Severely frail* | | 0.98 | 0.60 - 1.59 | 0.9 |
| **NLR (Ref = Low NLR)** | | - | - | - |
| *High NLR* | | 1.63 | 1.09 - 2.44 | 0.017* |
| **PLR (Ref = Low PLR)** | |  |  |  |
| *High PLR* | | 1.03 | 0.74 - 1.43 | 0.9 |
| **LMR (Ref = Low LMR)** | |  |  |  |
| *Low LMR* | | 1.52 | 1.11- 2.08 | 0.008* |
| **SIII (Ref = Low SIII)** | |  |  |  |
| *High SIII* | | 0.80 | 0.55-1.77 | 0.3 |
| **Variable** | | **Hazard Ratio (HR)***^1^* | **95% CI***^1^* | **p-value** |
| **Cancer-specific survival** | | | | |
| **Performance Status (Ref = PS 0)** | |  |  |  |
| *1* | | 2.13 | 1.39 - 3.25 | <0.001** |
| *2* | | 2.00 | 1.14 - 3.52 | 0.015* |
| *3* | | 0.63 | 0.08 - 4.77 | 0.7 |
| **Stage (Ref = Early)** | |  |  |  |
| *Advanced* | | 1.42 | 0.85 - 2.35 | 0.2 |
| **Subsite (Ref = Glottic)** | |  |  |  |
| *Supraglottic* | | 1.33 | 0.82 - 2.17 | 0.3 |
| *Subglottic* | | 4.53 | 1.01 - 20.3 | 0.049* |
| *Transglottic* | | 1.91 | 1.01 - 3.59 | 0.046* |
| *Indeterminate* | | 3.70 | 0.82 - 16.8 | 0.089 |
| **Nodal Status (Ref = N0)** | |  | - |  |
| *N1* | | 1.38 | 0.64 - 2.98 | 0.4 |
| *N2* | | 2.27 | 1.36 - 3.78 | 0.002* |
| *N3* | | 4.82 | 1.98 - 11.8 | <0.001** |
| **NLR (Ref = Low NLR)** | |  |  |  |
| *High NLR* | | 1.37 | 0.75 - 2.50 | 0.3 |
| **PLR (Ref = Low PLR)** | |  |  |  |
| *High PLR* | | 1.05 | 0.66 - 1.67 | 0.8 |
| **LMR (Ref = Low LMR)** | |  |  |  |
| *Low LMR* | | 1.79 | 1.17 - 2.73 | 0.007* |
| **SIII (Ref = Low SIII)** | |  |  |  |
| *High SIII* | | 0.95 | 0.54 - 1.68 | 0.9 |
| **Variable** | **Hazard Ratio (HR)***^1^* | | **95% CI***^1^* | **p-value** |
| **Recurrence-free survival** | | | | |
| **Performance Status (Ref = PS 0)** |  | |  |  |
| *1* | 1.71 | | 1.16 - 2.52 | 0.007* |
| *2* | 1.24 | | 0.69 – 2.21 - | 0.5 |
| *3* | 1.91 | | 0.45 – 8.11 | 0.4 |
| **Stage (Ref = Early)** |  | |  |  |
| *Advanced* | 1.04 | | 0.69 – 1.58 | 0.8 |
| **Nodal Status (Ref = N0)** |  | | - |  |
| *N1* | 1.13 | | 0.48 – 2.69 | 0.8 |
| *N2* | 2.27 | | 1.34 – 3.86 | 0.002* |
| *N3* | 3.17 | | 1.11 – 9.07 | 0.032* |
| **NLR (Ref = Low NLR)** |  | |  |  |
| *High NLR* | 1.01 | | 0.57 – 1.77 | 0.9 |
| **PLR (Ref = Low PLR)** |  | |  |  |
| *High PLR* | 1.09 | | 10.69 – 1.71 | 0.7 |
| **LMR (Ref = Low LMR)** |  | |  |  |
| *Low LMR* | 1.75 | | 1.15 – 2.67 | 0.009* |
| **SIII (Ref = Low SIII)** |  | |  |  |
| *High SIII* | 1.15 | | 0.67 – 1.99 | 0.6 |

*P values <0.05 were defined as significant (*), with P values <0.001 highly significant (**).*
